# Fossils from South China redefine the ancestral euarthropod body plan

**DOI:** 10.1186/s12862-019-1560-7

**Published:** 2020-01-08

**Authors:** Cédric Aria, Fangchen Zhao, Han Zeng, Jin Guo, Maoyan Zhu

**Affiliations:** 1 State Key Laboratory of Palaeobiology and Stratigraphy & Center for Excellence in Life and Palaeoenvironment, Nanjing Institute of Geology and Palaeontology, Chinese Academy of Sciences, Nanjing, 210008 China; 2 Management Committee of the Chengjiang Fossil Site World Heritage, Chengjiang, 652599 China; 3 College of Earth and Planetary Sciences, University of Chinese Academy of Sciences, Beijing, 100049 China

**Keywords:** Euarthropoda, Body plan, Cambrian, Chengjiang, Macroevolution, Homology

## Abstract

**Background:**

Early Cambrian Lagerstätten from China have greatly enriched our perspective on the early evolution of animals, particularly arthropods. However, recent studies have shown that many of these early fossil arthropods were more derived than previously thought, casting uncertainty on the ancestral euarthropod body plan. In addition, evidence from fossilized neural tissues conflicts with external morphology, in particular regarding the homology of the frontalmost appendage.

**Results:**

Here we redescribe the multisegmented megacheirans *Fortiforceps* and *Jianfengia* and describe *Sklerolibyon maomima* gen. et sp. nov., which we place in Jianfengiidae, fam. nov. (in Megacheira, emended). We find that jianfengiids show high morphological diversity among megacheirans, both in trunk ornamentation and head anatomy, which encompasses from 2 to 4 post-frontal appendage pairs. These taxa are also characterized by elongate podomeres likely forming seven-segmented endopods, which were misinterpreted in their original descriptions. Plesiomorphic traits also clarify their connection with more ancestral taxa. The structure and position of the “great appendages” relative to likely sensory antero-medial protrusions, as well as the presence of optic peduncles and sclerites, point to an overall homology with the anterior head of radiodontans. This is confirmed by our Bayesian phylogeny, which places jianfengiids as the basalmost euarthropods, paraphyletic with other megacheirans, and in contiguity with isoxyids and radiodontans.

**Conclusions:**

*Sklerolibyon* and other jianfengiids expand the disparity of megacheirans and suggest that the common euarthropod ancestor possessed a remarkable phenotypic variability associated with the externalized cephalon, as well as endopods that were already heptopodomerous, which differs from previous hypotheses and observations. These animals also demonstrate that the frontalmost pair of arthrodized appendage is homologous between radiodontans and megacheirans, refuting the claim that the radiodontan frontal appendages evolved into the euarthropod labrum, and questioning its protocerebral identity. This evidence based on external anatomy now constitutes a solid benchmark upon which we should address issues of homology, with the help of carefully examined palaeoneurological data.

## Background

Arguably the most successful animals, arthropods have an exceptionally versatile body plan whose origin is of great interest to evolutionary research as a whole. Since the discovery of the Burgess Shale, and later of other similar Cambrian Lagerstätten, notably those of South China, a wealth of non-biomineralized fossil species from the Cambrian have provided us with an invaluable insight into the diversity and morphological characteristics of early arthropods, commonly seen as fitting outside of the ‘great four’ traditional extant groups: Chelicerata, Myriapoda, and the paraphyletic Crustacea, which contains Hexapoda (together forming the Pancrustacea [1–3]). Recently, however, the idea that many of these taxa documented the earliest picture of euarthropod evolution has been challenged. Hymenocarines have been shown to be mandibulates [4], and perhaps even early pancrustaceans [5, 6], while more problematic species have been assigned to chelicerates [7] or the chelicerate stem [8], providing support to the Arachnomorpha hypothesis and thus the derived condition of trilobites and their relatives. Altogether, these reassessments emphasize the dramatic nature of the Cambrian explosion in the earliest Cambrian, implying also that we have far less evidence about the first true arthropods than we had thought. Or perhaps that some of this evidence has been overlooked, focused as we have been on fossils mistaken for representatives of incipient anatomies.

*Fortiforceps* and *Jianfengia* are rare early Cambrian arthropods known so far exclusively from the Chengjiang biota, China [9–11]. Because they characteristically bear a pair of prominent, dorsally-oriented frontal appendages composed of multiple articulating claws, they have been associated with other “great appendage” Cambrian arthropods—such as *Yohoia*, *Leanchoilia* and *Haikoucaris*—and placed with them in the class Megacheira [10]. Megacheirans have been thought to share the same simple body plan features: bipartite head-trunk tagmatization with overlapping and undifferentiated tergites; homonomous bipartite limbs with paddle-like exopod fringed with thin lamellae; and, importantly, four-segmented heads (also equivalent to five somites; see ref. [12] for a discussion on whether the ocular somite should be considered a segment). Together with the poorly known *Pseudoiulia* [13], the Chinese taxa *Fortiforceps* and *Jianfengia* have otherwise been diagnosed by slender, multisegmented bodies (with trunks composed of 20 somites or more) and, allegedly, multipodomerous limbs (composed of about 15 podomeres or more). This would contrast with the common seven-podomerous endopods and well-constrained number of trunk segments of other megacheirans, which are typified by only 11 to 13 segments [14, 15]. As a result, these megacheirans with fewer segments have been phylogenetically discriminated and placed within the clade Cheiromorpha [14], leaving Megacheira as a potentially paraphyletic group.

Following the recent phylogenetic reappraisals of hymenocarines and other taxa [4, 5, 8], megacheirans may be more pivotal to our understanding of euarthropod origins, since they could be the earliest arthropods with fully arthrodized bodies (i.e., whose segments are all articulated through arthrodial membranes). However, among megacheirans, the “multisegmented” species remain poorly known and the redescriptions of both *Fortiforceps* and *Jianfengia* have been lacking to this day. Because these species are thought to constitute derivatives or perhaps ancestral representatives of the better-known cheiromorph body plan [11, 14], they could be very relevant candidates to try and address questions relative to early morphological evolution in euarthropods. Their study is especially timely in light of recent advances in the understanding of radiodontans [16] and isoxyids [17], recognized as sister groups to Euarthropoda.

One of the most discussed topics in the recent palaeontological literature about arthropods is that of the homology of the various frontalmost appendages, and, incidentally, the anatomy of the head tagma. In the span of a few years, a series of papers documenting fossilized nervous tissues from the early Cambrian of China have laid out a homology map of important early arthropod groups [18, 19], shortly followed by extensive reviews of arthropod evolution based on these new findings [20]. According to these studies, the radiodontan frontalmost appendage would be innervated by the anteriormost part of the brain, the protocerebrum, and would be analogous to the deutocerebral great appendage of megacheirans. This view has been criticized [4, 5, 17], mostly because it is directly conflicting with evidence from external morphology in the fossil record, such as the presence in the isoxyid frontalmost appendage of both radiodontan and megacheiran characters, in addition to an apparent conservation of the head tagma between isoxyids and megacheirans. It is therefore crucial, as well as timely, to approach this issue with additional evidence if we are to understand arthropod origins.

Hereafter, we reexamine *Fortiforceps* and *Jianfengia*, as well as introduce a new genus, based on a variety of new and previously reported fossil material from the lower Cambrian Maotianshan Shale in Haikou, Kunming and Chengjiang, Yuxi, Yunnan.

## Results

For Systematic Palaeontology, see Additional file 1. Terminology follows ref. [14]. YKLP 11350, previously assigned to *Pseudoiulia cambriensis* [11], is here synonymized with *Sklerolibyon maomima* gen. et sp. nov.

### Habitus

*Fortiforceps*, *Jianfengia* and *Sklerolibyon* are small euarthropods (between ca. 1 and 4 cm) with prominent multichelate frontal appendages and elongate, slender multisegmented bodies bearing long biramous appendages.

### Frontal appendages

**Fig. 1:**
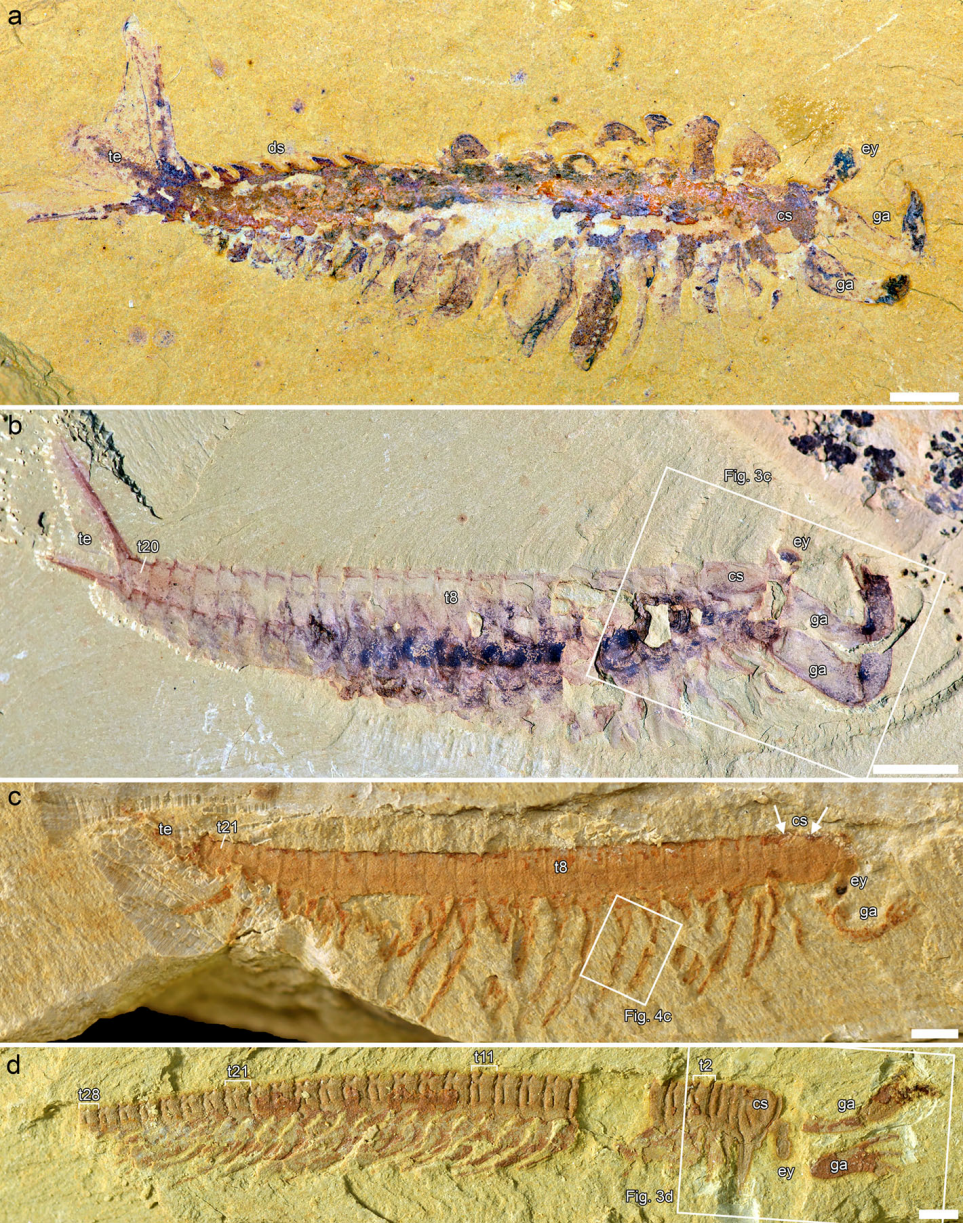
Jianfengiids, general anatomy. **a**, **b**, *Fortiforceps foliosa*. **a**, NIGPAS 169948, from Ercaicun. **b**, CJHMD 00020, from Heimadi. Inset is Fig. 3c. **c**, *Jianfengia multisegmentalis* NIGPAS 169957, from Mafang. Superimposed image of two photographs taken at different angles (emphasizing both relief and colouration). Inset is Fig. 4c. **d,** *Sklerolibyon maomima* gen. et. sp. nov. NIGPAS 169962, holotype, from Mafang. Inset is Fig. 3d. Arrows in c point to segmental impressions underneath the cephalic shield. All pictures taken in non-polarized light and dry. Scale bars: 1 mm (**c**, **d**); 2 mm (**a**); 5 mm (**b**)

**Fig. 2:**
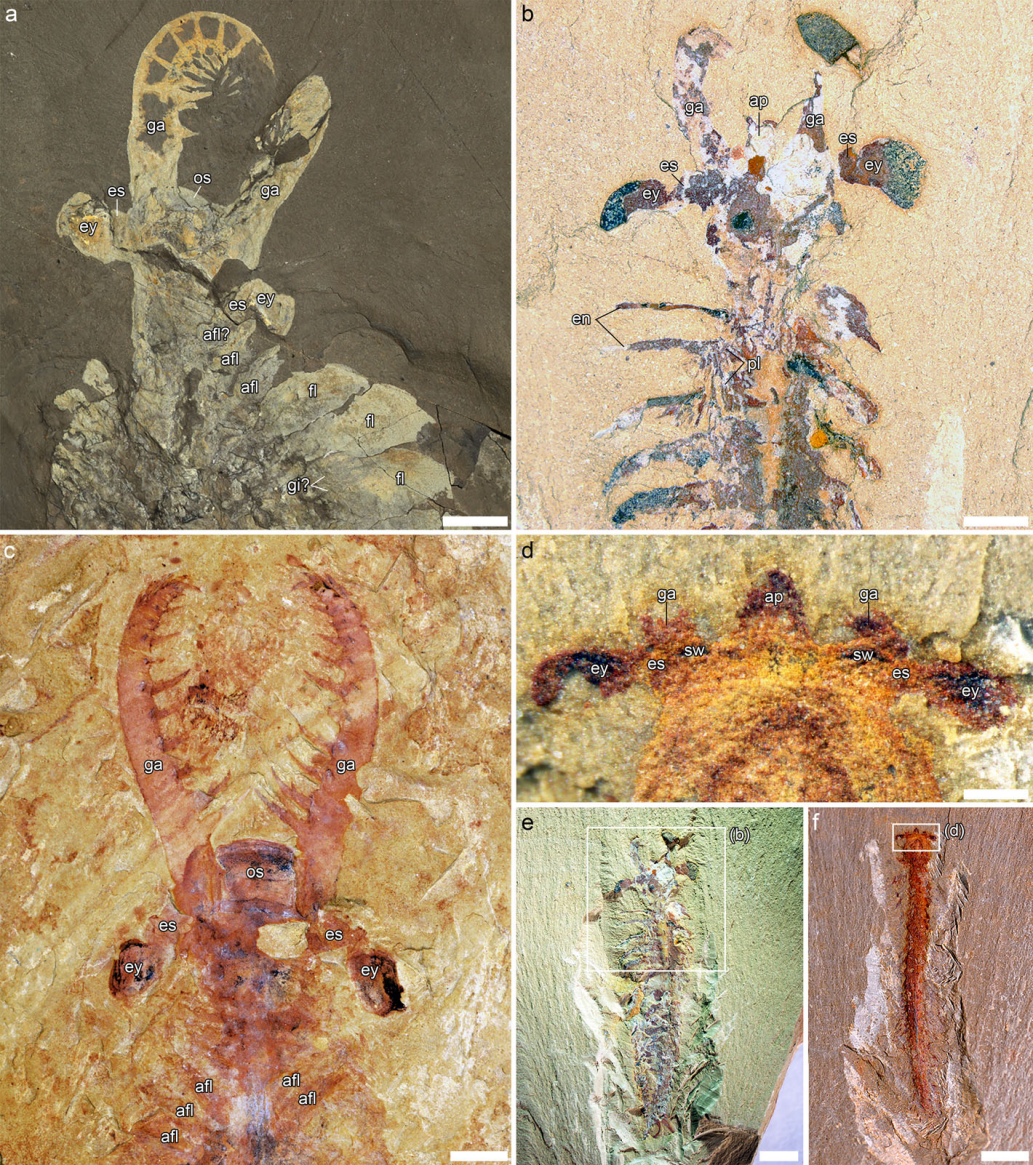
Anterior cephalic morphology of jianfengiids and comparison with *Anomalocaris*. **a**, *Anomalocaris canadensis* ROMIP 51212, from the Burgess Shale. **b, e,** *Fortiforceps foliosa* NIGPAS 169956, from Mafang. Inset in e refers to b. **c,** *Anomalocaris* sp. ELRC 20001, from Maotianshan. **d, f,** *Jianfengia multisegmentalis* NIGPAS 169959, from Mafang. Inset in f refers to d. All pictures taken in non-polarized light and dry, except for a, photographed wet. Scale bars: 0.25 mm (**d**); 1 mm (**b**); 2 mm (**e**, **f**); 5 mm (**c**); 20 mm (**a**)

**Fig. 3:**
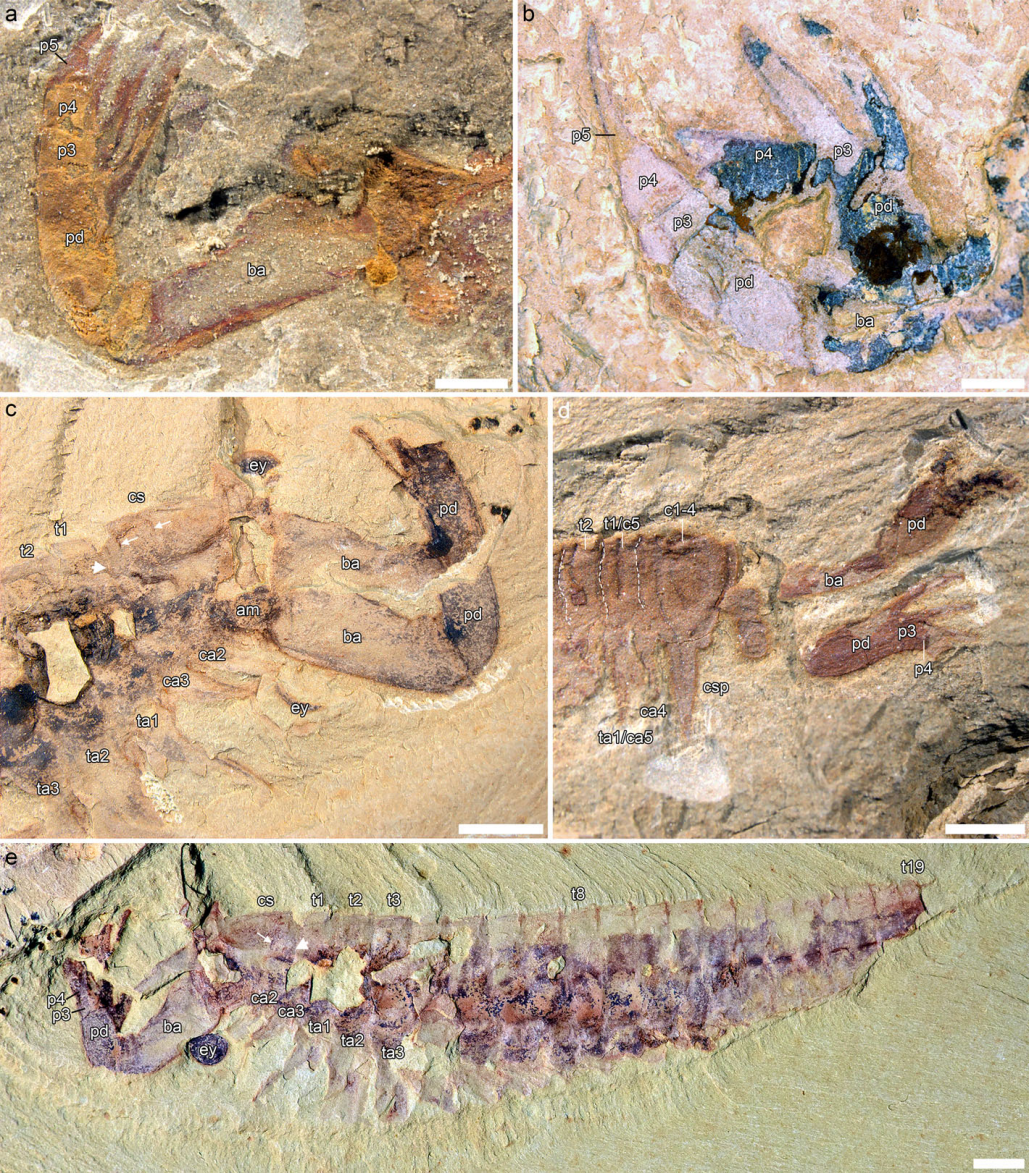
Head tagma and great appendages of jianfengiids. **a**-**c**, **e**, *Fortiforceps foliosa*. **a,** NIGPAS 169954, from Mafang. See also Additional file 1: Figure S1c. **b,** NIGPAS 169953, from Xiaolantian. **c, e,** CJHMD 00020, from Heimadi. See also Fig. 1b. **d,** *Sklerolibyon maomima* gen. et. sp. nov. NIGPAS 169962, holotype and only specimen, from Mafang. See also Fig. 1d. Small arrows in c and e indicate impressions of segmental boundaries on the cephalon; large arrowheads in c and e indicate the posterior margin of the cephalon; dashed lines in d mark the posterior margins of cephalic segments 2 and 3, and of the first trunk segment. All pictures taken in non-polarized light and dry. Scale bars: 1 mm (**a**, **b**, **d**), 2.5 mm (**c**, **e**)

The frontal “great” appendages of *Fortiforceps*, *Jianfengia* and *Sklerolibyon* share the same basic architecture (Figs. 1, 2b, 3, and 5, Additional file 1: Figure S1; see also ([11], fig. 5B): an elongate proximal podomere (basis), another elongate but stouter podomere (peduncle) attached to the basis at an angle and bearing the proximalmost and longest of the ventral spines (on its distal margin), followed by shorter podomeres bearing successively shorter (yet still elongate) ventral spines and ending in a fourth spine which forms a chelate device with the third one; all of these claws’ tips being thus aligned dorsally.

The great appendages of *Fortiforceps* are distinct in having a shorter and much more robust basal podomere than those of *Jianfengia* and *Sklerolibyon*, stout across all its length. *Jianfengia* and *Sklerolibyon* both have chalice-shape peduncles (Figs. 1c, d, 3d, 5, Additional file 1: Figure S1a, b, Figure S2a, b), contrasting with a more tubular shape in *Fortiforceps*, although they are distinctly slenderer in *Jianfengia* (Figs. 1c, Additional file 1 Figure S2) compared to the stout peduncles of *Sklerolibyon*. The distal claws appear thinner in *Jianfengia* and *Sklerolibyon* than in *Fortiforceps*. In light of this, the great appendages of *Jianfengia* and *Sklerolibyon* are structurally—and thus likely functionally—more similar to those of *Yohoia*, while the stouter form of *Fortiforceps* is rather akin to that of *Parapeytoia*. Although *Haikoucaris* possesses only three distal claws, it also has stronger peduncles, from which the leanchoiliid type of great appendage may be derived ([14]; our phylogeny in Fig. 5a).

A small proximalmost podomere basal to the elongate basis was reported present in *Yohoia* and influenced a specific hypothesis of podomere homology across great appendages [21], postulating a common bi-podomerous basis. We found a rounded element between the body and the elongate proximal podomere of the great appendage in *Fortiforceps* (Figs. 1b and 3c, e), but it does not bear the characteristics of a podomere with well-defined margins. It is more likely that this basalmost piece in *Fortiforceps* and other megacheirans with yohoiid-like great appendages is a relatively large arthrodial membrane serving as attachment to the body, whereas leanchoiliids possess bi-podomerous bases. *Haikoucaris* represents an intermediate condition with a three-clawed distal structure lacking flagellae and a bi-podomerous basis [22]. It follows that the number of segments of the yohoiid type of great appendages matches that of leanchoiliids, with the peduncle in the yohoiid type losing its spine and becoming a basal podomere in *Haikoucaris* and leanchoiliids (under the assumption that the yohoiid type is ancestral [14]).

### Eyes and features of the ocular region

In *Fortiforceps*, *Jianfengia* and *Sklerolibyon* the lateral eyes are ovoid to bean-shaped and distinctly pedunculated (Figs. 1, 2b, d-f, 3c-e, 5, Additional file 1: Figure S1a, d, Figure S2a, b, Figure S3a, b). No median eyes have been identified. *Fortiforceps* and *Jianfengia* also share a particularly well-developed medial protrusion aligned with the eye insertion (Fig. 2b, d, Additional file 1: Figure S3a, b; see also ([11], fig. 5c, left inset). Based on their anterior and pre-oral location, these protrusions are potentially homologous to the arthropod labrum, particularly similar to the way it is expressed in chelicerates, as discussed for other Cambrian taxa [4, 5]. At least in *Fortiforceps*, this frontal extension of the body is covered by a round sclerite, on the sides of which insert two elongate rami with clubbed terminations, as documented by the dorsally-preserved YKLP 11354 and YKLP 11355 ([11], fig. 7a, e). *Fortiforceps* and *Jianfengia* also display a pair of swellings on the eye stalks (Fig. 2b, d, Additional file 1: Figure S3a, b).

### Cephalic shield

The cephalic shield of *Fortiforceps* is distinct among megacheirans in that it covers the anterior portion of the body in a very tightly fashion. Hence the eyes, eye stalks and the base of the frontal appendages are usually visible (Figs. 1a, b, 2b, e, 3a, c, e and 5, Additional file 1: Figure S1d, f). Most striking is the absence of pleural extensions on the cephalon, when they are characteristically well developed in cheiromorphs [14, 21–23]. Along the trunk, segmental boundaries are clearly preserved, while the posterior cephalic margins are visible in the holotype (see ref. [10] fig. 31A) and in CJHMD 00020 (Fig. 3c, e), and therefore a distinct, articulated and sclerotized cephalon is likely present, as opposed to having a radiodontan-like condition. This morphology stands out as possibly intermediary, derived from a hardening of the radiodontan head cuticle with little to no separation of the shield’s margins from the head proper. Faint traces of segmental boundaries are still visible in the sclerotized head and correspond to cephalic somites 2/3 and 3/4 (Fig. 3c, e; see below).

By contrast, *Jianfengia* possesses a more distinct head shield with respectively rounded and semi-circular outlines in dorsal and lateral view (Figs. 1c and 2f, Additional file 1: Figure S2, Figure S3a). The lateral aspect is reminiscent of bivalved arthropod carapaces, particularly of *Surusicaris*, an isoxyid [17]. Individuals can preserve the impression of three underlying segmental units under the shield (Fig. 1c). The holotype [24, 25] and “*Pseudoiulia*” YKLP 11352 [11] display a pair of triangular processes between the eyes, which are most likely absent in at least NIGPAS 169957 (Fig. 1c) and NIGPAS 169961 (Additional file 1: Figure S3). Two morphs of *Jianfengia multisegmentalis* are therefore known based on cephalic morphology (overlapping with trunk polymorphism; see below).

*Sklerolibyon* bears a heavily-sclerotized head shield distinct in turn from both *Fortiforceps* and *Jianfengia* (Figs. 1d and 3d, Additional file 1: Figure S1a; see also ([11], fig. 5B). A large and straight, gladius-like spine adorns the antero-lateral margins. The fused first three tergites are delimited by cuticular borders running along the front and occipital margins and lateral spines. An additional segment smaller than trunk ones appears to complete the head tagma, and is fully differentiated externally.

### Head tagmata and post-frontal appendages

*Fortiforceps* has a set of three biramous limb pairs posterior to the great appendages that seem discretely smaller than the remaining posterior pairs (Figs. 1b, 3c, 5). A broadened base in these limbs likely corresponds to the basipod, but no podomere boundary is clearly preserved in these limbs. Of these three smaller pairs, only the first two belong to the cephalon, as shown by the position of the first trunk tergite (Figs. 1b, 3c, 5). This is confirmed by unarticulated segmental impressions in the head.

Impressions of segment boundaries in *Jianfengia* divide the cephalon into three distinct units (Fig. 1c, Additional file 1: Figure S2). The frontal unit houses the ocular and great appendage somites. Three pairs of smaller appendages (compared to trunk ones) are otherwise present under the cephalic shield (Additional file 1 Figure S2a-c; see also ([25], fig. 20.17a-c). Remarkably, and consistently across most specimens (Additional file 1: Figure S2a, b; see also ([25], fig. 20.17a-c), an additional pair of appendages with length similar to those of the trunk insert at the occipital margin of the cephalic shield, that is, at the boundary between the head and trunk tagmata. For simplicity and consistency with cheiromorphs, we start counting trunk segments from this appendage pair, but it could also be considered as a fifth cephalic segment. This does not appear to be the case in the smaller CJHMD 0022 from Heimadi, in which the first pair of longer appendages belong to a distinct post-cephalic segment (Additional file 1: Figure S2c), but other specimens of comparable size also possess a transitional limb pair (e.g. holotype NIGPAS 10012). Exopods on the anterior limbs appear to be present on the holotype [24]. In CPS 1611, a couple of anterior elements that could be mistaken for antennular appendages are in fact remains of the post-frontal endopods from the left side of the animal (Additional file 1: Figure S2b).

The externalized head of *Sklerolibyon* is similar to that of *Jianfengia*, but the first trunk segment is well expressed and seems partially fused to the head shield. Only two pairs are visibly associated with the cephalon in NIGPAS 169962 (Figs. 1d and 3d, Additional file 1: Figure S1a), but YKLP 11350 ([11], fig. 5B) displays three distinct and small endopods within the cephalic area, as well as an additional appendage inserted at the posterior margin of a modified trunk-like segment interpreted as the first segment of the trunk (see also Additional file 1). The length of this intermediate pair is not known. We construe that the two remaining pairs in NIGPAS 169962 are hidden by the cephalic shield.

Jianfengiids therefore exhibit two different types of cephalons: one which encompasses strictly three segments (or four somites), and one that is made of 4 + 1 segments (or 5 + 1 somites), considering that one limb pair inserts at the head-trunk boundary. Both situations differ from that of cheiromorphs, which are united by four-segmented cephalons.

### Trunk

We have identified two morphs of *Fortiforceps foliosa* for trunk segment number. Most specimens match the holotype CN 115372 in having 20 trunk segments, but NIGPAS 169961 and F006 from Mafang clearly have 22 trunk segments. This suggests the existence of another species, also supported by the thicker telson rods and more pronounced dorsal spines in these specimens, but the evidence remains too scarce overall to fully justify the formal erection of a new taxon at this time—ELRC20501 from Maotianshan, for instance, shows morphological characteristics similar to those of Mafang individuals, but the exact number of trunk segments is impossible to determine. Segments are homonomous and characteristically bear tooth-like projections on their latero-dorsal margins, oriented latero-dorsally; these spines become increasingly sharper posteriad, taking on the shape of curved blades. The pleural extensions of the segments are not well known, but they seem to simply enclose the body latero-ventrally, possibly joining with sternites. Posterior tapering starts to be visible after segment 11 and is very gradual as well as limited in extent; posteriormost segments are more than half the length of anterior ones.

Interestingly, *Jianfengia multisegmentalis* also comprises morphs with 20 and 22 trunk segments (including the holotype IGP 100123, in which the telson head was originally counted as the posteriormost segment), as well as another large morph with 27, represented by CSP 1611. The majority of specimens also belong to the morph with 20-segmented trunk. *Jianfengia* specimens possess a distinct telson head in the shape of a right-angle triangle that should not be confused with a posteriormost trunk segment (Additional file 1: Figure S2 ). Additionally, NIGPAS 169961 shows that *Jianfengia* had a subtle but intricate articulation pattern between trunk segments (Additional file 1: Figure S3). The posterior margin of at least the anterior trunk segments is “collar-like,” producing short and rounded pleural extensions on each lateral side (ra1, Additional file 1: Figure S3e, f). The anterior circumference of the segments is marked by the presence of two thin and separate ridges (rp1 and rp2, Additional file 1: Figure S3e, f). An additional section of cuticle at the point of articulation forms a strip that seems to belong to the previous segment and represent the real line of segmental division (ra2, Additional file 1: Figure S3e, f). This is suggested by the fact that, posteriad, ra1 becomes thinner and the deepening following ra2 becomes more pronounced. In posteriormost segments, only the depression at ra2 remains visible (Additional file 1: Figure S3h). These thin ridges are not cuticular folds per se, because their pattern is regular, but they could represent lines of greater flexibility of the cuticle, possibly related to an early stage of body arthrodization. The articulation of the cephalic shield with the first trunk segment appears to be made out of a similar cuticular pattern.

*Sklerolibyon* has a very long 34-segmented trunk (Fig. 1d, Additional file 1: Figure S1a, b; see also ([11], fig. 5B). Each junction between segments bears an elevated cuticular half-ring, and each segment bears an additional half-ring located at 2/5th of the segment’s length from its posterior margin. In addition to the same features present in the “head,” this forms a series of 71 thickened rims along the body. This configuration shows similarities with the intricate nature of the inter-segmental morphology in *Jianfengia* (Additional file 1: Figure S3).

*Jianfengia* bears very short, rounded pleurae on its trunk segments (Additional file 1: Figure S3). Specimens of *Sklerolibyon* do not preserve outstanding pleurae, but we construe that they are likewise very short.

*Internal organs*. *Fortiforceps* specimens display—across a range of preservation quality—sets of three-dimensional structures (sometimes due to phosphatization) at the base of trunk limbs (Additional file 1: Figure S1f). These structures are elongate and radiate from a single point or short line, forming bundles of filaments or lamellae. We assume for now that these features are very proximal lamellae of the exopod paddle, but they could also be separate gill-like elements as was illustrated for *Alalcomenaeus* [17]. The gut in *Fortiforceps* usually preserves as a longitudinal dark stain on the specimens, without clear indication of stomach or diverticula. However, there are sub-triangular projections overlapping the base of the limbs that match those previously described in arthropods from the Burgess Shale and interpreted as hemocoelic cavities (Fig. 3c).

Internal tissues do not preserve well in *Jianfengia*, except for features of the ocular area in NIGPAS 169959 and NIGPAS 169961. No internal feature is known in *Sklerolibyon*.

### Trunk limbs

**Fig. 4:**
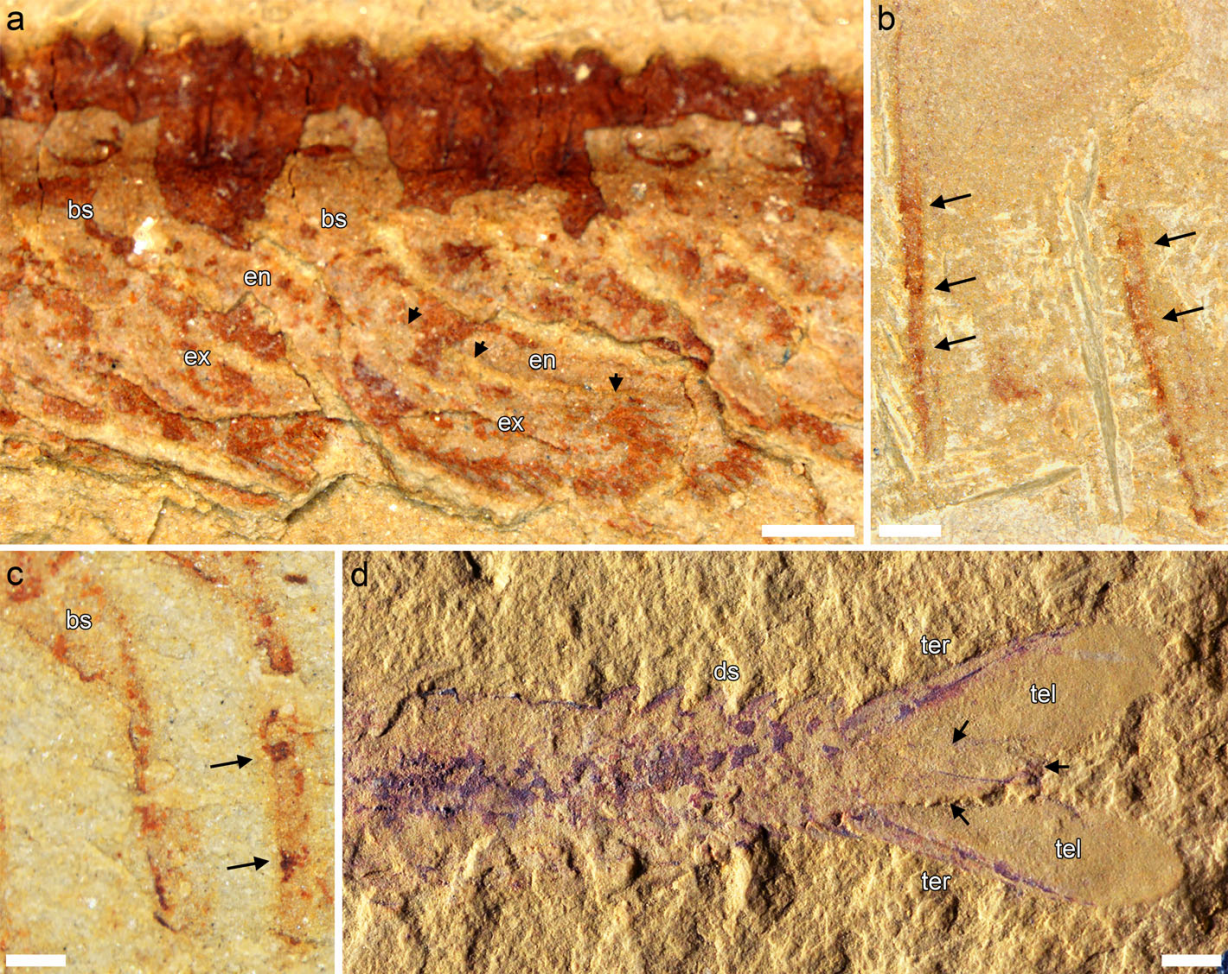
Details from appendage and tailpiece morphology in jianfengiids. **a**, *Sklerolibyon maomima* gen. et. sp. nov. NIGPAS 169962, holotype and only specimen, from Mafang, close-up of trunk midsection. See also Additional file 1: Figure S1b. **b, d,** *Fortiforceps foliosa*. **b,** NIGPAS 169954, from Mafang, close-up of trunk endopods. See also Additional file 1: Figure S1c. **c,** *Jianfengia multisegmentalis* NIGPAS 169957, from Mafang, close-up of trunk endopods. See also Fig. 1c. **d,** NIGPAS 169949, from Ercaicun, posterior end including tailpiece. Arrows in a points to dorsal margin of endopod; in **b** and **c**, to podomere boundaries; and in **d**, to lateral margin of the medial element within the tailpiece. All pictures taken in non-polarized light and dry. Scale bars: 0.5 mm (**a**-**c**), 1 mm (**d**)

**Fig. 5:**
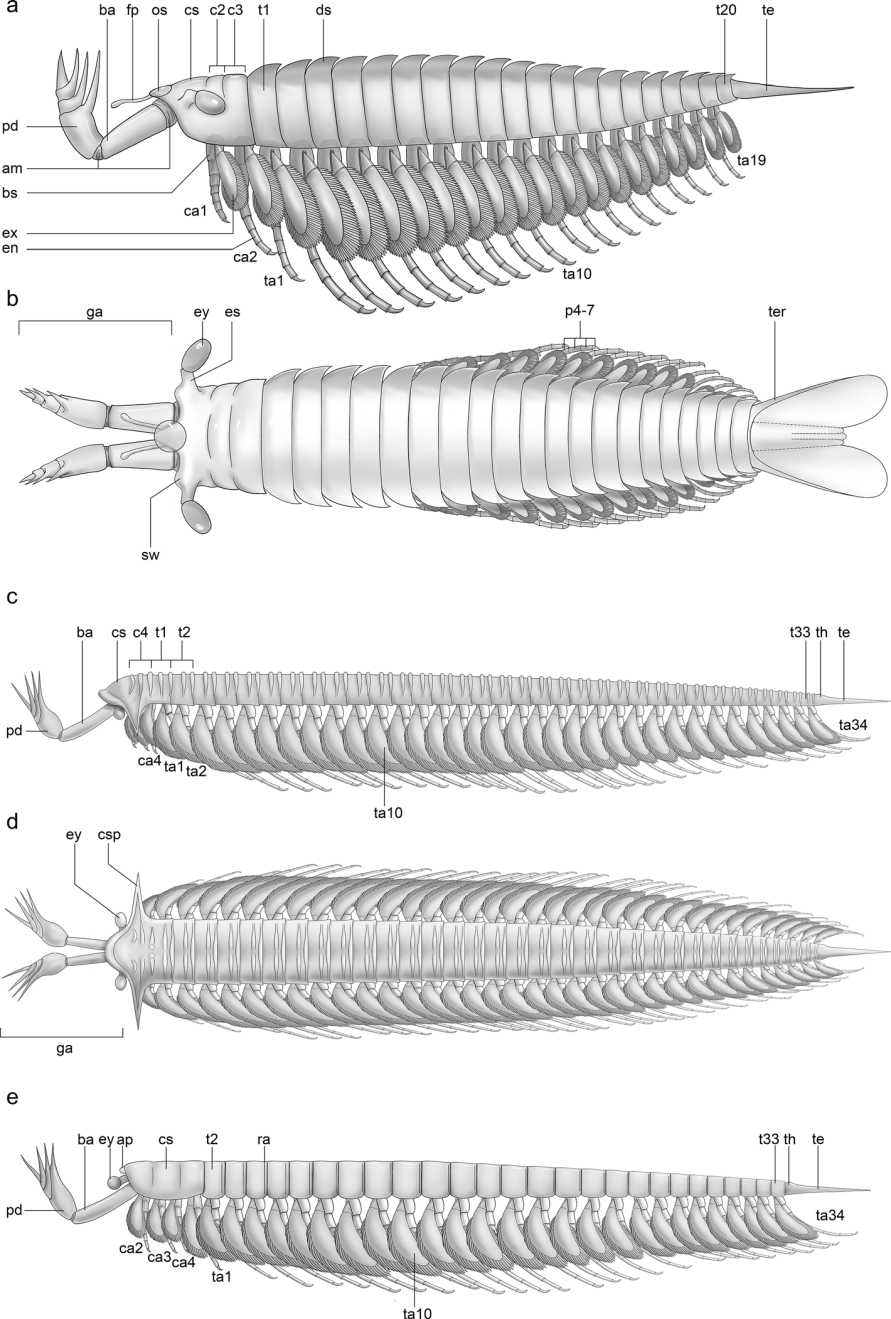
Diagrammatic reconstructions of jianfengiids **a**, **b**, *Fortiforceps foliosa*, lateral and dorsal views, respectively. **c**, **d,** *Sklerolibyon maomima* gen. et sp. nov, lateral and dorsal views, respectively. The telson of *Sklerolibyon* is hypothesized based on the morphology of *Jianfengia*. **e,** *Jianfengia multisegmentalis*, morph from Daipotou with 27 trunk somites, lateral view. Drawings by Dinghua Yang

*Fortiforceps*, *Jianfengia* and *Sklerolibyon* have one limb pair/somite per segment. Only trunk segment 20 in *Fortiforceps* seems to be limbless. The first trunk pair is consistently shorter (by about 1/5th up to 1/3rd of length when measurable) than posterior trunk limbs (Figs. 1b, 3c, e, 5, Additional file 1: Figure S2). Endopods are long and slender, composed of elongate podomeres bearing at least a pair of small distal spines (Fig. 4a-c). An accurate podomere count per endopod is not possible with our material, but an an heptopodomeran condition is likely based on NIGPAS 169954 (Additional file 1: Figure S3d). Exopods are typically megacheiran, that is, paddle-shaped and fringed with oblanceolate lamellae. They are however more elongate in *Fortiforceps*, *Jianfengia* and *Sklerolibyon* than in other megacheiran taxa, tending to have a more oblong shape. The type of attachment of the exopod on the basipod is not known, but we assume here it is similar to that of other megacheirans.

Basipods in the examined taxa are broad, subtriangular in lateral view and show no evidence of differentiation (Figs. 1c, 3c, e, 4a and 5, Additional file 1: Figure S2a). Subdivisions and presence of endites are unknown.

### Tailpiece

In *Fortiforceps*, two highly sclerotized and spike-like, carinate rods or ‘beams’ forming a 55 to 85-degree angle constitute the armature of the tailpiece. A wider but much thinner and roughly rectangular structure projects medially and forms the center of the tailpiece. A thin and deformable membrane is attached to the entire length of both lateral beams and connects to the medial structure. In EC 15000, the membrane forms a single concave posterior margin overlapping the medial structure (Fig. 1a). However, they were originally described from CN 115373 [10] to form two lobes extending beyond the tips of the lateral rods before joining up with the postero-lateral ends of the medial structure—as can be faintly seen also in NIGPAS 169949 (Fig. 4d). The latter lobate configuration seems to correspond to narrower tailpieces in which the lateral beams are separated by a smaller angle. If the widening of the tailpiece was structurally possible, the lobate margins of the flexible membrane would indeed be stretched out during that process, possibly adopting the concave shape otherwise observed. The medial sub-rectangular element was originally described in detail as a set of three lobate flaps with separate distal tips, based exclusively on CN 115373. There are also traces of what seems to be separate elements at the tip of the medial structure in NIGPAS 169948, but we find no compelling additional evidence in our other specimens.

Although heavily modified for swimming, the tailpiece of *Fortiforceps* technically remains a telson, because the lateral rods are fused at their base, which represents a post-segmental extension of the body.

We do not have dorso-ventral specimens of *Jianfengia* preserving the tailpiece very clearly, but it seems from NIGPAS 169958 and CJHMD 00021 that it is an elongate, spine-shaped telson (see Additional file 1: Figure S2).

### Phylogeny

Our Bayesian analysis retrieves both Megacheira and Cheiromorpha as paraphyletic groups (Fig. 5a). Multisegmented megacheirans, or jianfengiids, as studied here—*Jianfengia*, *Fortiforceps* and *Sklerolibyon*—form a clade basal to cheiromorphs and all other euarthropods. *Parapeytoia* resolves as a strong sister genus to *Fortiforceps*, but this is likely the result of a biased optimization of the many uncertain character states coded for this taxon (see Additional file 1). The exclusion of *Parapeytoia* does not otherwise affect jianfengiid relationships (topology not shown). The presence of masticatory gnathobases and the lack of elongate endopod podomeres as in other Jianfengiidae suggest a more derived placement of *Parapeytoia*, closer to chelicerate and artiopodans. *Jianfengia* resolves as the most basal of jianfengiids, and thus as the basalmost euarthropod. There would be reasons to think that *Fortiforceps* should fulfill that role (e.g., shorter head shield, rounded antero-medial sclerite), but polarization conflicts explain this result (see below).

The morphological dataset used here recently provided support for the monophyly of Arachnomorpha [8], but the inclusion of the taxa presented in this study seems to favour instead Antennulata, that is, the grouping of Artiopoda and Mandibulata (Fig. 5a). As a result, Arachnomorpha apomorphies (viz. tripartite apotele and gnathobasipods, see ref. [8]) would become synapomorphies for all non-megacheiran euarthropods (that is, here, crown-group euarthropods). This highlights the enduring nature of the problematic phylogenetic resolution of trilobites and their relatives, either with respect to other euarthropod clades or internally. Higher nodes within Artiopoda are also arranged differently compared to recent restudies of this group [26, 27], in spite of the overlap of most characters between the two datasets. In both analyses, using a Bayesian approach, these higher nodes are weakly supported.

## Discussion

### The question of the antennules

*Fortiforceps* was originally described [10] as having a pair of antennules (or “first antennae”) in front of the great appendages, based notably on CN 115375. This would imply that great appendages are homologous to the post-frontal equivalents in extant taxa—antennae in crustaceans and pedipalps in chelicerates, but others have considered that these appendages are anterior to the antennules and derived from the ocular somite [28]. An assortment of arthropod remains from the Stanley Glacier site of the Burgess Shale placed under the name *Kootenichela deppi* [29], allegedly showed, similar to *Fortiforceps*, faint traces of antennules, in addition to the presence of great appendages. However, the interpretation was considered problematic [17], with the various specimens probably belonging to different taxa and the holotype itself possibly being a bivalved arthropod instead (CA, pers. obs.). More recently, new *Fortiforceps* specimens were documented that showed more convincingly the presence of antero-medial projections with elongate stems and club-like tips [11], as originally described. However, there is no decisive argument to homologize these structures with mandibulate antennules. Rather, their association with a likely ocular sclerite and their inter-ocular location suggest an origin within the ocular somite, and hence a homology with similar structures in *Canadaspis* [4, 30] and other early panarthropods, although these protocerebral sensory devices display an evolutionary pattern strongly driven by convergence or parallelism [31]. The great appendages clearly insert posteriorly to these projections and any associated sclerite and thus likely belong to a posterior somite. *Jianfengia* specimen CPS 1611 shows some traces of cuticle above the great appendages, but these are remains of cephalic limbs on the left side of the body (Additional file 1: Figure S2b).

### Topological identity of the frontalmost appendage

**Fig. 6:**
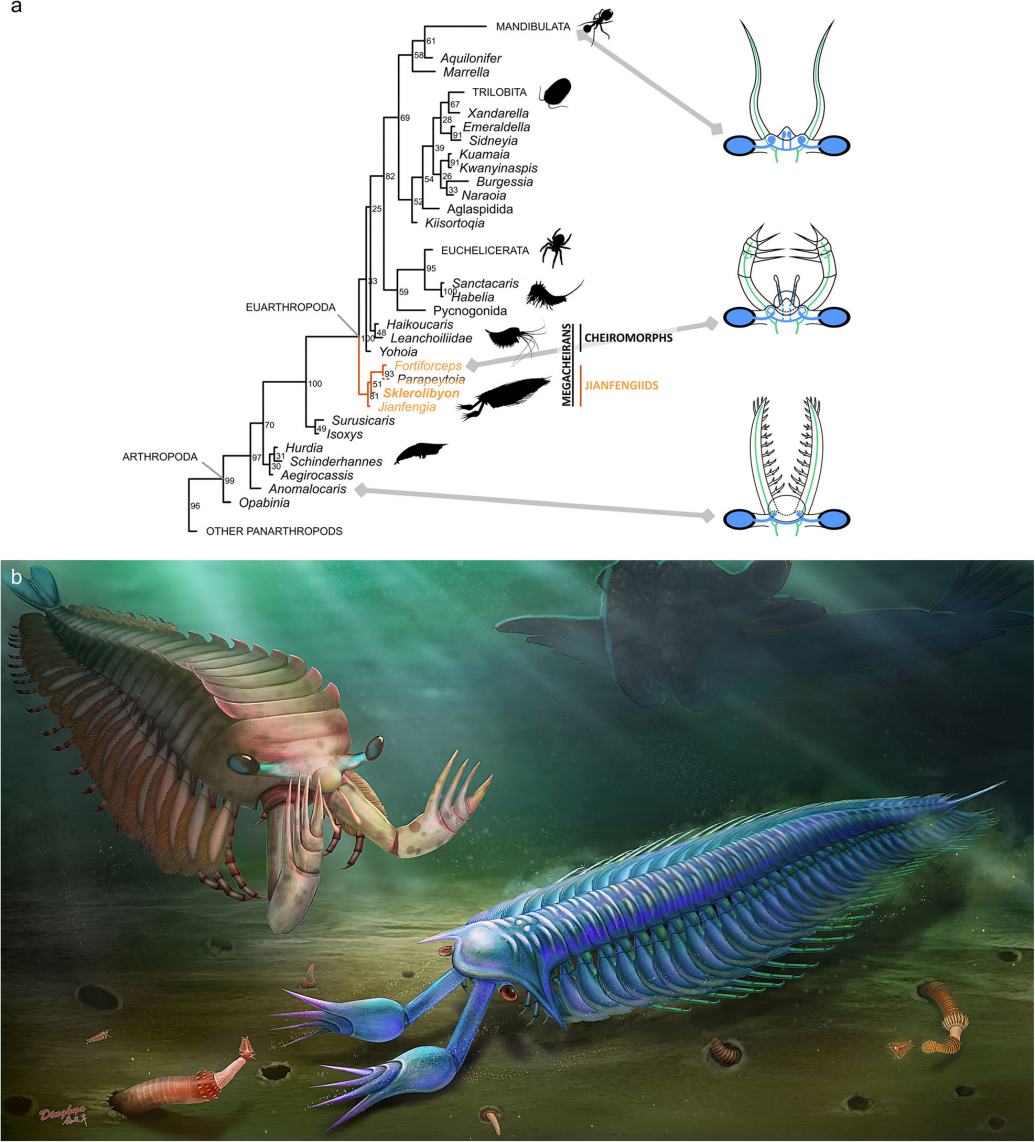
Evolutionary implications of the jianfengiid anatomy and artistic reconstructions of *Fortiforceps foliosa* and *Sklerolibyon maomima* gen. et sp. nov. in early Cambrian Gondwanian seas. **a**, left: Simplified consensus cladogram showing the position as earliest euarthropods of *S. maomima* gen. et sp. nov. and other jianfengiids in the panarthropod evolutionary tree. The position of *Parapeytoia* is considered doubtful and the result of a methodological limitation (see main text and Supplementary Text); Right: Head diagrams of a generalized anomalocaridid (bottom), a *Fortiforceps*-like jianfengiid (middle) and a waptiid hymenocarine (top), showing topological relationships between cephalic features, notably frontal appendages, anterior sensory organs and eyes. In blue are neural features corresponding to the protocerebrum, and in green those of the deutocerebrum; we thus represent here a hypothesis of ancestral deutocerebral homology of the frontalmost arthrodized appendage. **b,** drawing by Dinghua Yang

We show here that the frontal regions of radiodontans and jianfengiids are nearly identical in topology and morphology, with the attachment of their frontalmost arthrodized appendages aligning comparably with the position of the pedunculate eyes and frontal structures (Fig. 6). Their respective frontalmost arthrodized appendages are therefore most likely homologous, consistent with previous hypotheses based on external morphology [17, 21, 22, 28, 32].

A recent line of evidence considered to support the analogy (rather than homology) between radiodontan and euarthropod frontalmost appendages came from palaeoneurological data and, prominently, the description of radiodontan brain morphology [19]. However, in light of our finding, if the interpretations of *Lyrarapax*’ alleged neural tissues were correct, then the megacheiran frontal appendage would not be deutocerebral, but protocerebral. This is in direct conflict with another interpretation of neural tissues in a leanchoiliid taxon from the same group of authors favouring a deutocerebral identity [18]. These interpretations of putative neural tissues are therefore internally inconsistent and must be critically reevaluated.

The description of neural tissues in *Lyrarapax* relies on a single specimen which shows, not nerves as they are known to preserve in Burgess Shale-type deposits (BST; i.e., filament-like extensions [5, 14, 33]), but paired spots, more reminiscent of other paired sensory organs documented in early arthropods [4, 5, 8, 34]. It should also be considered that these spots could be part of the inter-ocular bridge, given their alignment with the base of the ocular peduncles (see structures on each side of ‘frg’ in their fig. 2). In Tanaka et al. [18], we believe the problem comes from an overconfident interpretation of internal tissue preservation in BST deposits. Although it is possible to discriminate internal tissues topologically when they are exposed individually, there is no method or rationale published to date to identify different tissues within a compressed patch of kerogen—through concentration of iron, copper or otherwise [35]—, and it is therefore not possible to clearly distinguish what is nerves and ganglions, digestive system, coelomic infill or other tissues in their fossil. As a result, the conclusions of these studies are questionable (see Additional file 1 for complementary discussion.)

Recently, the Sirius Passet lobopodian *Kerygmachela* has been redescribed and medial reflective remains from new fossil material have been interpreted as elements of the CNS [36]. While this seems likely the case given the topology and morphology of the tissues, we do not think that it is possible to constrain the homology of the various brain neuropils as the authors do—especially to ascertain the identity of the main pair of nerves as protocerebral. Other interpretations are equally plausible. As a matter of fact, the configuration documented—a pair of large latero-frontal nerves and smaller frontalmost projections—is also found in larval sea spiders, which bear reduced ocular nerves frontally and chelifore nerves at the antero-lateral extremities of the stomodeal ring [37]. Despite such frontal location, chelifores have been found to be associated with the deutocerebral neuropil [38]. It is thus not possible to validate a protocerebral identity of *Kerygmachela*’s frontal appendages at present. More generally, the fact that the brain ganglia in adult arthropods are often fused into a coherent mass called the syncerebrum [39] make their visual discrimination difficult, which is why neurological studies generally use early instars (e.g., [40]).

Topological arguments have also been put forward to justify a different segmental origin between radiodontan and megacheiran frontalmost appendages [20], all related to the insertion of appendages relative to the mouth. However, steps leading to a postero-ventral opening with rotation of the oesophagus (characteristic of more derived euarthropods) from a simpler ventral aperture are not well known. Ventral documentation of frontalmost appendage insertion relative to the mouth opening is lacking in adult megacheirans, except for a specimen of *Yawunik* suggesting, in fact, that the great appendages were juxtaposed anteriorly to the stomodaeum [14], much as they are thought to be in radiodontans. In addition, a postero-ventral opening seems already present in *Opabinia* [41]. Even if a small topological difference in oral connection existed, it would not justify advocating the analogy of two similarly structured frontalmost arthrodized appendages within a similar anterior anatomical context and between two phylogenetically contiguous sets of taxa.

The presence of inter-ocular, non-podomerous sensory projections in *Fortiforceps* also constrains the identity of the great appendages, because these projections appear more likely to originate from the protocerebrum. The great appendages would therefore belong to the posterior neuropil, which, by definition, is the deutocerebrum. This contradicts the hypothesis that radiodontan frontal appendages evolved by reduction into the euarthropod labrum, either by positing their homology between radiodontans and megacheirans [28], or by considering them to be respectively proto- and deutocerebral in origin [20]. A much better candidate for the ancestral labrum is the pre-oral frontal projection documented here, for instance, in jianfengiids, and taking a variety of forms in early arthropods [4, 8], as well as seemingly taking the shape of a more conventional labrum in some larvae [42]. Given that the labrum is now well documented to be both protocerebral and appendicular in origin [43, 44], it should be considered whether inter-ocular projections such as those of *Fortiforceps* or *Canadaspis* have any connection to labral origins, despite their scattered presence along the arthropod tree [31]. The presence of separately differentiated protocerebral organs in radiodontans (as, possibly, in *Lyrarapax* [19], see Additional file 1) could further the view that anterior protocerebral appendages may have been co-opted very early on as frontal sensory structures precursors to the labrum in panarthropods.

Hymenocarine anterior anatomy [4, 5], in general, supports a homology between early arthropod great appendages and antennules. *Waptia*, for instance, shows a similar frontal organization with a central “labral complex” adjacent to pedunculate eyes under which both antennules are inserted [5] (Fig. 5a). In addition, both hymenocarines and habeliidans display very anterior mouth openings with associated frontal labral-like organs [4, 8], which also favours a conservation of the radiodontan general anteriormost anatomy rather than a sudden and drastic reorganization with posteriorization of the labrum.

Because mandibulate antennules and chelicerae likely belong to the deutocerebral segment [12, 40, 45] (but see ref. [46]), the de facto protocerebral innervation of onychophoran antennae [47], placed basally in the panarthropod tree, remains an argument that a change of frontalmost appendage from proto- to deutocerebrum possibly occurred at some point during the evolution of this group (irrespective of additional protocerebral features). *Aysheaia* [48], which frontally bears antennae similar to those of onychophorans, while also linking them morphologically (through the presence of ventral processes) with the frontalmost appendages of ‘large lobopodians’ (e.g. *Kerygmachela* and *Megadictyon*), may represent the strongest connecting fossil evidence for an ancestral protocerebral identity of this appendage, in direct conflict with the points raised above.

It should be perhaps considered that the antennae of onychophorans are not, in fact, homologous to the frontalmost appendages of *Aysheaia* and/or large lobopodians. Although, in this case, external morphology could argue for topological identity, a considerable evolutionary gap does in fact separate onychophorans from lobopodian ancestors, involving terrestrialization and important changes in head anatomy, not the least being the full ventralization of the mouth, associated with a complex reorganization of its innervation [49] (*contra* the claims in ref. [20], all early lobopodians, including *Hallucigenia*, likely had a terminal mouth [50]). The presence of one or two pairs of ‘antennae’ in *Antennacanthopodia* [51] in addition to a pair of lobopods associated with the ‘head’ well supports the relative plasticity of protocerebral appendages in the total-group Onychophora [50]. It has also been argued that genetic evidence for homology between the onychophoran antennae and the arthropod labrum should be interpreted with caution in light of the complexity of genetic networks [52].

Regardless of neural identity, the strongly-supported homology between the frontalmost arthrodized appendages of radiodontans (or dinocaridids), isoxyids [17, 32] and megacheirans should be used as a solid benchmark to discuss head evolution in early arthropods. It also follows that the hypothesis of transformation of the radiodontan frontalmost appendage into the labrum is rejected, along with evolutionary scenarios heavily based on this idea.

### “‘*Yohoia*-type’ great appendage”

The great appendages of *Fortiforceps*, *Jianfengia* and *Sklerolibyon* belong with those of *Yohoia* and *Parapeytoia* to the same morphological ‘*Yohoia*-type’ sub-group: these are constituted of an elongate basal podomere articulating via an “elbow” with a peduncle and three podomeres that bear four long and robust distal claws.

Previous alignments [21, 22] of megacheiran appendages are contested by the evidence presented here. Our material shows that there is no podomere proximal to the elongate podomere we call here the basis, but rather what seems to be a large arthrodial membrane. Such membrane is also present at the base of at least some anomalocaridids appendages, as in *Amplectobelua* [53]; a soft structure striated with membranous folds (fig. 4 in the cited study) was likewise misinterpreted as a basalmost podomere in this taxon, due its deceptively large size. There thus seems to be a one-to-one equivalent between the podomeres of multisegmented megacheirans’ great appendages and those of cheiromorphs’, with the peduncle of the latter being the second spinose podomere of *Yohoia*-like forms.

Biomechanically, the *Yohoia*-type appendage mostly relied on its single basal segment and strong arthrodial membrane to project the distal claws and then retract it along with the prey towards the body, as was described before based on a two-podomerous basis [21]. We do not know if this movement was fast, like in mantis shrimps, but the prey’ integument was most likely soft, owing to the absence of strong gnathobases in these taxa. In *Fortiforceps* and possibly *Parapeytoia*, the basal segment is distinctly stouter, suggesting that the appendage must have been capable of holding larger prey relative to body size.

### Inter-ocular region

At least *Fortiforceps* and *Jianfengia* among jianfengiids bear well-developed antero-medial protrusions (and, in the former, associated sensory projections) that are likely related to a variety of such features among arthropods, especially Cambrian taxa [4, 8, 10, 34]. The condition in jianfengiids is similar to that of relevant artiopodans [10] (e.g. *Kuamaia* and *Saperion*), hymenocarines [4, 5, 34] or even *Anomalocaris* [54], which all possess an antero-medial complex formed of a separate sclerotic element often housing or covering fleshy, sensory organs. Following the hypothesis formulated in the case of habeliidans [8], this structure in jianfengiids could represent the plesiomorphic condition of the labrum, especially in its chelicerate form ([55]; see above).

The presence of differentiation of the eye stalks (‘peduncular lobes’) is also remarkable, because it is potentially associated with the development of large neuropils housed by these swellings. In *Waptia*, it was discussed how the location of these lobes was consistent with that of olfactory organs [5]. This could set the origin of these organs (at least in a well-developed form) deep within Euathropoda.

### Ancestral head tagma

It has been proposed recently that the ancestral “two-segmented head” defended by some authors [20, 28, 56] was a biased interpretation based on the difficulty of accessing evidence for other cephalic appendages in fossil taxa whose head is extensively covered by carapaces [4, 5]. Instead, the ancestral four-segmented (pentasomitic) head tagma advocated by Walossek and Müller [57] could be a more accurate representation of the fossils evidence [4], as illustrated by *Surusicaris* [17] and cheiromorphs [21, 22]. The possibility of having three pairs of differentiated flaps behind the frontalmost appendage or other types of somites contributing to a cephalic tagma has also been discussed in radiodontans [53, 54].

The conditions seen in *Fortiforceps*, *Jianfengia* and *Sklerolibyon* impose a reevaluation of that concept. If, developmentally, the head tagma was already composed of five somites in isoxyids and perhaps radiodontans, the respectively less and more inclusive cephalons of *Fortiforceps* and *Jianfengia* / *Sklerolibyon* suggest an ancestral plasticity in the formation of head shields between closely related taxa. The corollary is that tergal tagmatization of the head may have been initially decoupled from its somitic tagmatization, the head shield being occasionally formed as a shorter unit than the developmental head, as defined by homeotic and gap genes during ontogeny [45]. It is also possible that the cephalon arose secondarily and sequentially, starting with the integration of two post-frontal segments in *Fortiforceps*, followed with at least one additional segment in *Sklerolibyon*, *Jianfengia* and cheiromorphs. Although certain features, such as the rounded anterior sclerite and the poorly individualized head shield, point to a basalmost position for *Fortiforceps* within jianfengiids, this is currently not directly supported by our phylogenetic results (Fig. 6). The basal placement of *Fortiforceps* in connection with the morphology of anomalocaridids is hampered by the intermediary position of isoxyids, which, given our current understanding [17, 32], do not show these specific comparable features, but other plesiomorphic traits (e.g., tail flaps, lobopodous limbs, dorsal spines on frontal appendage). Such an alternative result is also mitigated by the paraphyly of radiodontans and the derived placement of hurdiids in this topology. This apparent conflict calls for further investigations of the isoxyid and radiodontan head anatomy.

### Trunk morphology in jianfengiids

*Fortiforceps*, *Jianfengia* and *Sklerolibyon* show a surprising variability in trunk morphologies compared to other megacheirans. The cephalic spines and complex segmental armature of *Sklerolibyon* are unique within this group, and suggest that megacheiran body plans might have been more disparate early in their radiation. Cuticular differentiations are also remarkable in the megacheiran-like *Tanglangia* [11, 58, 59], but some features (e.g., elongate telson, 13-segmented trunk) suggest it may rather be transitional between cheiromorphs and habeliids, at the base of the panchelicerate lineage.

Trunk pleurae in *Jianfengia* and *Sklerolibyon* are very short, giving their segments a more ring-like shape, as is known notably in hymenocarines. *Fortiforceps* possesses a more rectangular cross-section, with pleurae seemingly lacking any free component and composing in their entirety lateral “body walls” below the rows of dorso-lateral spines.

These paired dorsal spines in *Fortiforceps* are also remarkable. Although dorsal projections are also known in *Habelia*, for instance, they are here distinctly tooth- to blade-like, consistently adorn each segment and project dorso-laterally instead of dorsally. It is noteworthy that features of nearly the exact same shape and topology were documented in the anomalocaridid *Aegirocassis benmoulae* and described as dorsal flaps [16]. Their likely presence in *Peytoia* [16] supports the view that these elements might have been originally soft (like flaps), but the morphology of *Fortiforceps* may question whether they were appendicular in origin, or if they were instead cuticular elements derived from an ancestral form of externally expressed segments. This alternative interpretation would be more in line with the hypothesis that biramicity originated from the subdivision of an existing limb axis, as suggested by the morphology of *Surusicaris* [17].

The fact that *Jianfengia* specimen CPS 1611 has a greater number of segments and is also larger than other specimens could suggest that different ontogenetic stages with anamorphic or hemianamorphic growth are present. The observed variation in the individualization of the first trunk segment can support this view. However, CPS 1611 is also from a distinct locality (Dapotou) and the degree at which variations in the expression of the head shield are affected by taphonomy is unclear. It is therefore difficult to decide at present whether these variations are simply polymorphic, ontogenetic, inter-specific or even sexual. This also applies to the anterior cephalic spines present in the holotype, although, in this case, an inter-specific difference seems more likely.

### The significance of trunk limbs

The endopods of *Fortiforceps* were initially described as ‘multipodomerous,’ [10] consisting of “about 15 [quadrate] podomeres.” This is not the case. In both *Fortiforceps* and *Jianfengia*, the podomeres are elongate and most likely cylindrical, implying that they are much fewer per endopod than originally thought. Our material suggests that a heptopodomeran condition (i.e., seven podomeres, see ref. [14]) is likely. The endopods of jianfengiids would be therefore structurally similar to those of cheiromorphs, differing mainly by the presence of elongate podomeres which give a long and slender aspect to the appendages (Fig. 6).

## Conclusions

Because the type and extent of body and post-frontal limb arthrodization in isoxyids remain unclear, multisegmented megacheirans, first described more than 30 years ago and now grouped within the family Jianfengiidae fam. nov., represent the earliest unequivocal euarthropods—as defined by the presence of fully arthrodized bodies and limbs [60] (Fig. 5b). This has the following implications for the origin of Euarthropoda:

- The plesiomorphic cephalic shield incorporated only three segments (four somites) in at least some of the earliest euarthropods, although the developmental head tagma could have been made of four segments (five somites). The externalization of the head tagma into a head shield therefore appears to have been particularly flexible in the common euarthropod ancestor. This cephalic shield could have been formed originally through an individualization of the radiodontan head cuticle (as seems to be the case in *Fortiforceps*) or from an equivalent of the isoxyid carapace (as suggested by *Jianfengia*), with which it would share a developmental origin;
- The frontal, raptorial great appendage of the earliest euarthropods is inherited from radiodontan and isoxyid ancestors and is therefore plesiomorphic for Euarthropoda. These limbs did not evolve into the labrum, which rather originated from antero-medial organs with an initially sensory function and innervated by the protocerebrum. This clarification now allows for a correct reading of macroevolutionary studies, such as disparity analyses [17]. Radiodontan frontalmost appendages, through intermediate isoxyid morphologies, therefore evolved into a set of specific raptorial appendages, multi-chelate, upward-directed and made of few podomeres—suggesting the existence of strong ecological factors of directional selection;
- Consequently, a parsimonious reading of brain homology, considering the current consensus on the frontalmost (arthrodized) appendage neural identity in extant taxa [12], would be that this appendage has a deep deutocerebral origin, at least from radiodontans onward;
- In association with “great appendages,” radiodontans and jianfengiids also share antero-medial, inter-ocular structures including body protrusions and sensory organs that were sometimes covered by an isolated sclerite. These features are likely inherited from lobopodians and persist in more derived euarthropods at various degrees. Their probable protocerebral identity and the existence of appendicular outgrowths, such as in *Fortiforceps*, designate them as strong candidates for the origin of the euarthropod labrum, in particular in the panchelicerate lineage.
- The presence of elongate podomeres and probably the heptopodomeran condition of endopods are anchored as a plesiomorphy of Euarthropoda, with no known intermediary to this date.

## Methods

### Fossil material

Our study material comprised 10 specimens of *Fortiforceps foliosa* (NIGPAS 169948–169,956, ELRC 20501 and CJHMD 00020), seven specimens of *Jianfengia multisegmentalis* (NIGPAS 169957–169,961, CPS 1611, CJHMD 0021 and CJHMD 0022) and one specimen of *Sklerolibyon maomima* gen. et. sp. nov. (NIGPAS 169962). All fossils specimens studied here are housed at the Nanjing Institute of Geology and Palaeontology, Chinese Academy of Sciences (NIGPAS), with the exception of with the exception of CJHMD 00020, CJHMD 00021 and CJHMD 00022, housed at the Chengjiang Fossil Museum of the Management Committee of the Chengjiang Fossil Site World Heritage, China. All fossils come from the Maotianshan Shale Member of the Yu’anshan Formation (Cambrian Series 2, Stage 3, *Eoredlichia*-*Wutingaspis* Trilobite Assemblage Zone), the typical fossiliferous interval of the Chengjiang Lagerstätte. Specimens were collected from different localities in Haikou, administrative division of the Xishan district, city of Kunming, and Chengjiang County, Yunnan Province, China. Those include Ercaicun (NIGPAS 169948, NIGPAS 169949, NIGPAS 169950, NIGPAS 169951, NIGPAS 169952) and Mafang (NIGPAS 169954, NIGPAS 169955, NIGPAS 169956, NIGPAS 169957, NIGPAS 169958, NIGPAS 169959, NIGPAS 169960, NIGPAS 169962) in Haikou, and Dapotou (CPS1611), Maotianshan (ELRC20501), Jiucun (NIGPAS 169961), Heimadi (CJHMD 00020, CJHMD 00021, CJHMD 00022) and Xiaolantian (NIGPAS 169953) within Chengjiang county. Detailed stratigraphic information and localities can be found in ref. [61].

### Preservation

As is generally the case for BST deposits, and in particular for Chengjiang-type early Cambrian Chinese Lagerstätten, fossils are preserved as largely pyritized aluminosilicates representing weathered metamorphic coatings of carbonaceous films and calcium phosphate precipitates [62]. All specimens are preserved in a latero-dorsal aspect, which is a common taphonomic characteristic of non-flat arthropods in BST deposits, save for three which are preserved in dorsal aspect.

### Observations

Specimens were observed and photographed in plain light with a Zeiss Discovery V16 microscope. A Nikon D800s fitted with a Nikon AF-S Micro Nikkor 105-mm lens was used to photograph larger specimens in full view. Images were then processed using Photoshop to adjust tone, color, contrast and brightness. Terminology used follows ref. [60].

### Phylogenetic analysis

The dataset is composed of 100 taxa and 267 discrete characters (Additional files 2 and 3), all subsequently unordered and unweighted, and with Priapulida used as outgroup. Bayesian phylogenetic analyses were performed using MrBayes [63] v.3.2.6 × 64. Tree searches followed an Mkv + Γ model [64] with four chains sampling during four runs for 10,000,000 MCMC generations, a tree sampled every 1000 generations and burn-in of 20%. A partial backbone constraint was used to enforce the monophyly of 15 extant clades based on separate morphological and molecular results (see Additional file 2). These clades were chosen as the highest nodes best supported by molecular or morphological data impossible to code in fossils, based mostly on a consensus of refs. [1–3, 65].

### Institutional abbreviations

CJHMD, Chengjiang County Museum of the Management Committee of the Chengjiang Fossil Site World Heritage; CPS, The Chengjiang Field Station of Palaeontology; NIGPAS or IGP, Nanjing Institute of Geology and Palaeontology.

## Supplementary information

**Additional file 1 Figure S1.** Anatomical comparison between *Sklerolibyon maomima* gen. et sp. nov. and *Fortiforceps foliosa*. a, b, *Sklerolibyon maomima* gen. et sp. nov. NIGPAS 169962, holotype, from Mafang. a, counterpart (negative imprint), with posterior end missing, shown here after applying inversion of light patterns, in order to show the positive and natural ornamentation. See also Fig. 1d. b, part (positive) with anterior section missing. Inset is Fig. 4a. c, YKLP 11350, paratype, close-up of the anterior region showing limb arrangement; note occasional overlap of endopods from both sides (double arrows). d, e, f, *Fortiforceps foliosa*. d, e, NIGPAS 169954, from Mafang. d, whole body. Insets are e, Fig. 3a and Fig. 4b, as indicated. e, close-up of endopod, showing a likely heptopodomerous condition. See also d. f, NIGPAS 169955, from Mafang, whole body; arrowheads point to partially phosphatized filamentous structures at the base of trunk limbs. All pictures taken in non-polarized light and dry. Scale bars: 1 mm (a, b), 2 mm (c, d). **Figure S2.** Segmentation patterns in *Jianfengia multisegmentalis*. a, b, CPS 1611, from Dapotou. a, Whole body, reconstructed from graphically adding the counterpart (posterior end) to the part (anterior end). b, Close-up of the cephalon, showing great appendages and both pairs of biramous cephalic limbs, from both left and right sides of the animal. c, NIGPAS 169958, from Mafang, whole body. d, CJHMD 0022, from Heimadi, whole body. e, CJHMD 0021, from Heimadi, whole body. All pictures taken in non-polarized light and dry. Scale bar: 1 mm. **Figure S3.** Morphological details in *Jianfengia multisegmentalis*. NIGPAS 169961, from Jiucun. a, Whole body. b, Close-up of anterior portion of head, showing anterior margin of cephalic shield and anteriormost body morphology. c, Close-up of biramous trunk limbs. d, Close-up of posterior end of body, showing posterior trunk limbs spread out. e-h, Close-ups of trunk sections, from anterior to posterior, showing details of intersegmental articulation. Arrowheads in b point to anterior margin of cephalic shield. All pictures taken in non-polarized light and dry. Scale bars: 1 mm (a, c, d), 0.5 mm (b, e-h). **Figure S4.** Consensus cladogram of a Bayesian analysis (Mkv + Γ model) of panarthropod relationships. Numbers next to nodes are posterior probabilities.

**Additional file 2.** mrb file including morphological matrix and parameters used for the phylogenetic analysis.

**Additional file 3.** List of characters used in our morphological matrix.

## Data Availability

All data generated or analysed during this study are included in this published article and its additional files.

## Abbreviations

afl: Anterior flap(s)
am: Arthrodial membrane
ap: Anterior protrusion
ba: Basis
bs: Basipod
ca*n*: Cephalic appendage* n*
c*n*: Cephalic segment *n*
cs: Cephalic shield
csp: Cephalic spine
ds: Dorsal spine(s)
en: Endopod
es: Eye stalk
ex: Exopod
ey: Eye(s)
fl: Flaps
fp: Frontal projections
ga: Great appendage(s)
gi: Gills
os: Ocular sclerite
pd: Peduncle
p*n*: Podomere *n*
ra1 and ra2: Anterior ridges 1 and 2
rp1 and rp2: Posterior ridges 1 and 2
sw: Swelling
ta*n*: Trunk appendage *n*
te: Telson
tel: Telson lobe
ter: Telson rod
th: Telson head
t*n*: Trunk segment *n*
