# Supplementary material for: Fossils from South China redefine the ancestral euarthropod body plan
Source: BMC Evol Biol. 2020 Jan 8;20:4. doi: 10.1186/s12862-019-1560-7 (PMC6950928; doi:10.1186/s12862-019-1560-7)
Supplement: Supplementary file 1 — Additional file 1 Figure S1. Anatomical comparison between Sklerolibyon maomima gen. et sp. nov. and Fortiforceps foliosa. a, b, Sklerolibyon maomima gen. et sp. nov. NIGPAS 169962, holotype, from Mafang. a, counterpart (negative imprint), with posterior end missing, shown here after applying inversion of light patterns, in order to show the positive and natural ornamentation. See also Fig. 1d. b, part (positive) with anterior section missing. Inset is Fig. 4a. c, YKLP 11350, paratype, close-up of the anterior region showing limb arrangement; note occasional overlap of endopods from both sides (double arrows). d, e, f, Fortiforceps foliosa. d, e, NIGPAS 169954, from Mafang. d, whole body. Insets are e, Fig. 3a and Fig. 4b, as indicated. e, close-up of endopod, showing a likely heptopodomerous condition. See also d. f, NIGPAS 169955, from Mafang, whole body; arrowheads point to partially phosphatized filamentous structures at the base of trunk limbs. All pictures taken in non-polarized light and dry. Scale bars: 1 mm (a, b), 2 mm (c, d). Figure S2. Segmentation patterns in Jianfengia multisegmentalis. a, b, CPS 1611, from Dapotou. a, Whole body, reconstructed from graphically adding the counterpart (posterior end) to the part (anterior end). b, Close-up of the cephalon, showing great appendages and both pairs of biramous cephalic limbs, from both left and right sides of the animal. c, NIGPAS 169958, from Mafang, whole body. d, CJHMD 0022, from Heimadi, whole body. e, CJHMD 0021, from Heimadi, whole body. All pictures taken in non-polarized light and dry. Scale bar: 1 mm. Figure S3. Morphological details in Jianfengia multisegmentalis. NIGPAS 169961, from Jiucun. a, Whole body. b, Close-up of anterior portion of head, showing anterior margin of cephalic shield and anteriormost body morphology. c, Close-up of biramous trunk limbs. d, Close-up of posterior end of body, showing posterior trunk limbs spread out. e-h, Close-ups of trunk sections, from anterior to posterior [file 12862_2019_1560_MOESM1_ESM.pdf]

Fossils from South China redefine the ancestral euarthropod body plan

Cédric Aria, Fangchen Zhao, Han Zeng, Jin Guo and Maoyan Zhu

SUPPLEMENTARY TEXT

Systematic palaeontology
Superphylum Panarthropoda Nielsen, 1995
Phylum Euarthropoda Lankester, 1904
Clade Heptopodomera Aria, Caron and Gaines, 2015

Class Megacheira Hou and Bergström, 1997
Diagnosis (emended from Hou and Bergström, 1997). Heptopodomeran euarthropods with the following characters: large and prominent pair of multi-chelate frontalmost appendages made of five podomeres; a simple cephalic shield covering two to three pairs of appendages with little to no morphological differentiation for food processing; series of homonomous trunk tergo-pleurae folded around the sub-cylindrical body; simple biramous trunk appendages with non enditic heptopodomerous endopods, robust basipods, and paddle-shaped exopods fringed at least medio-distally with contiguous oblanceolate lamellae. Tailpiece a telson.
Remark. We reuse the Class Megacheira to simplify the diagnosis of our new genus, but the monophyly of this taxon remains uncertain (see discussion herein on phylogenetic results). We consider that *Parapeytoia* should belong to this group and thus leave open any statement about the differentiation of basipods, in this case as masticatory gnathobases.

Family Jianfengiidae Aria, Zhao, Zeng, Guo and Zhu, fam. nov.
LSID: urn:lsid:zoobank.org:act:732C92A5-E42E-4019-B9AC-C4D6D72CFB88
Diagnosis. Megacheirans with the following characters: lateral eyes pedunculate; head shield formed by at least the first four anterior somites; well-marked traces of segmental boundaries in head shield; multisegmented trunk (segment number ≥20); trunk appendages long: exopod elongate, endopods made of elongate podomeres.
Type genus. *Jianfengia* Hou, 1987.
Other included taxa. *Fortiforceps foliosa* Hou and Bergström, 1997; *Sklerolibyon maomima* gen. et. sp. nov. Possibly includes *Parapeytoia yunnanensis* Hou et al., 1995.
Remark. It is uncertain whether *Parapeytoia* should belong to this family. The presence of *Fortiforceps*-like frontal appendages combined with many morphological uncertainties (e.g. head anatomy, number of trunk segments) is optimized in our phylogeny as an inclusion of *Parapeytoia* within Jianfengiidae with strong support. However, the presence of masticatory gnathobases and the lack of elongate endopod podomeres suggest this taxon is more derived, perhaps sister to Arachnomorpha (sensu Aria 2019). To clarify this, a reexamination of the holotype and additional specimens would be much needed. We note that Hou and Bergström (1997) erected the monospecific family Fortiforcipidae and order Fortiforcipida based on *Fortiforceps foliosa*. These taxonomic ranks are redundant and were not justified with respect to close megacheiran relatives, including the previously described *Jianfengia*. We therefore do not make use of them in this study.

Genus *Sklerolibyon* Aria, Zhao, Zeng, Guo and Zhu, gen. nov.
LSID: urn:lsid:zoobank.org:act:5D61B1E9-B95A-42CB-A9CA-070D1321A236

Etymology. From the Greek *σκληρός* (sklērós) and *λίβυον* (líbyon), meaning respectively “hard” and “wild lotus,” owing to the flower-shaped frontal appendages and armoured body of this fossil arthropod from South China.
Type and only species. *Sklerolibyon maomima* (see below).
Diagnosis. Jianfengiid megacheiran with the following characteristics: Body length 25 mm (without frontal appendages and telson); frontalmost appendages with long (ca. twice as long as anterior section of head shield), curved basal podomere and stout chalice-shaped peduncle; short armoured head shield representing the fusion and compression of the first three segments (or first four somites) and part of segment 4; antero-lateral margins of head shield produced into prominent gladius-shaped lateral spines; segment 5 shorter than trunk segments but with similar dorsal armature, seemingly also fused to the anterior shield; appendages of segment 5 biramous, likely smaller than further posterior appendages; appendages of segments 2-4 smaller than appendages of segment 5, seemingly 2/3rd of length of cephalic spine; trunk 33-segmented; each trunk segment divided out by two cuticular half-rings: one of these elevated cuticular ridges placed posteriorly at the points of articulation and contiguous with adjacent segments, while the other divides the segment transversally into a longer anterior part and shorter posterior part; intermediary half-ring with medial effacement interrupting the closure of the ring, although said effacement is somewhat attenuated in posteriormost segments; trunk pleurae likely short and little developed.
Remark. The delimitation of the head tagma is somewhat ambiguous, because segment boundaries are confounded with their marginal armature, also present in the head shield. The fifth pair of cephalic appendages and corresponding segment seem to be part of the cephalon, which would therefore result in a five-segmented head tagma. The posterior margin of the “genal” spines of the head shield, however, are located within segment 4, which would imply an anatomy that is more consistent with the known megacheiran body plan. For simplicity and to facilitate comparison with *Jianfengia*, we consider segment 5 as part of the trunk (but see main text).

Sklerolibyon maomima Aria, Zhao, Zeng, Guo and Zhu, sp. nov.
Figs. 1c, d, 3d, Extended Data Fig. 1a, b
LSID: urn:lsid:zoobank.org:act:466152EC-35DD-4073-AD94-62E471E8AD61
Synonymy
“Pseudoiulia cambriensis”, 2016 Strausfeld et al. p. 160, fig. 5B

Etymology. A combination of the Chinese 矛 (Máo) and the Latin “mimus,” together meaning “spear-like,” owing to the elongate and tapering antero-posterior silhouette of the animal, with long and adorned limbs giving a feathery aspect not unlike the collar of ancient Chinese spears.
Holotype. NIGPAS 169962.
Other material. YKLP 11350.
Occurrence. Locality of Mafang, Haikou A.D., district of Xishan, city of Kunming, Yunnan Province, China, within the Maotianshan Shale Member of the Yu’anshan Formation (Cambrian Series 2, Stage 3, *Eoredlichia-Wutingaspis* Trilobite Assemblage Zone).
Diagnosis. As per genus.
Description. *Habitus and tagmatization*. Small (25 mm without frontal appendages and tailpiece), very elongate and multisegmented arthropod with biramous limbs and prominent, raptorial frontal appendages. Body composed of 39 somites arranged into a five-somitic head and a 34-somitic trunk. Well-developed lateral spines on the anterior portion of the cephalic

shield and markedly reinforced trunk segments. Pleurae poorly developed, body a narrow, posteriad-tapering tube with sub-quadratic section.
Frontalmost appendages. Five-podomerous "great appendages" of the yohoiid type, i.e., ending in four imbricated podomeres produced ventro-distally into elongate spines. Basis (podomere 1) very elongate (width ca. 1/5th of length of frontal head shield; length 4/3rd of that of frontal head shield), slightly curving dorsally. Peduncle (podomere 2) remarkably large, chalice-shaped, articulated with the basis so that the multi-chelate portion of the appendage is oriented dorsally; ventro-distal spine likely aligned with spine tips of more distal podomeres, reaching ca. the same length as the peduncle, i.e., 2/3rd of basis length.
Eyes and inter-ocular area. One pair of well-developed, pedunculate lateral eyes. Eyes large. elliptical (major axis slightly inferior to half of cephalic width [without lateral spines]), protruding latero-frontally beneath head shield. Peduncles short. Median structures and presence of median eyes unknown.
Cephalon. Head tagma encompassing the first five somites. Tergites of segments 1 to 3 (=somites 2 to 4) fused into a ventrally bent almond-shaped shield, with long (ca. equal to width of shield proper) gladius-like spines produced on each side. Tergite of segment 4 expressed as a pseudo-segment with first half integrated to the anterior, spine-bearing portion of the head shield, and second half contiguous with the posterior margin of the cephalic spines and the anterior margin of segment 5. The frontal shield is shaped by a complex set of grooves and ridges: a frontal lip is followed centrally by a depression marking the anterior limit of the shield's dome; laterally, the frontal rim extends into the elevated medial axis of the lateral spines, and projects continuously behind the central dome to form the occipital margin of the frontal shield; an additional, less pronounced depression precedes the occipital lip of the shield. At a distance approximately equal to 1/5th of the frontal shield, posterior to the occipital lip of this shield, is another ridge delimiting the posterior margin of segment 4 (somite 5); posterior to this ridge lies segment 5, delimited posteriorly by a more or less rounded transverse carina and with an intermediate carina akin to those of trunk segments but only slightly closer to the posterior margin.
Cephalic appendages. Appendages of segment 5 biramous and likely similar in structure to trunk appendages, although seemingly shorter by a fourth or a fifth of their length. Appendages of segment 4 about 2/3rd shorter than appendages of segment 5; proximally, the limb bears a typically enlarged and undifferentiated basipod, and likely also an exopod. The exact podomere count for cephalic endopods is unknown; however, in comparison to what is known in *Jianfengia*, *Fortiforceps* and other megacheirans, we construe that, in the head, endopods of at least segment 4 are likely heptopodomerous.
Trunk. Post-cephalic tagma 34-segmented. Trunk segments are delimited by carinate half-rings at the points of articulation. Additionally, each trunk segment is divided into asymmetric anterior and posterior halves by another half-ring; anterior segmental interval ca. 2.5 times length of posterior part in anterior segments, and reduced to less than twice in posteriormost segments; medial effacement of the intermediary half-rings from the back of the frontal shield to last trunk tergite interrupting the closure of the rings dorsally, although said effacement is attenuated in posteriormost segments. Trunk pleurae likely short and little developed, with body forming a narrow, posteriad-tapering tube with sub-quadratic section.
Trunk appendages. Trunk appendages of general megacheiran structure: biramous, with large undifferentiated basipod bearing elongate endopods and paddle-shaped exopods fringed with likely oblanceolate lamellae. Endopods and exopods remarkably long and elongate, with endopods reaching up to four times the height of the corresponding segment, and exopods up to four times as long as wide. Whether the basis is composed of a single podomere is unknown; exopod attachment also unknown.
Tailpiece. Tailpiece unknown.

Remark. YKLP 11350 was previously assigned to *Pseudoiulia cambriensis* Hou and Bergström, 1998 [3]; however, *Pseudoiulia* has wide tergites with short but well-developed pleurae and segments lack strong ornamentation [2, 4]. YKLP 11350 instead presents all the characteristics of *Sklerolibyon maomima* as described above, including the characteristic segmental ornamentation. YKLP 11352 in ref. [3] was also referred to as *Pseudoiulia*, but the original documentation is insufficient to confidently identify this specimen. Based on the presence of anterior cephalic spines (see main text), it would seem to belong instead to *Jianfengia*.

Owing to the very small size of the last visible segments on the part of the holotype, we consider very likely that they terminate the body and that the posteriormost piece of cuticle broken through by the fracture in the sample is in fact part of the telson. Thus the total somite count is probably accurate, despite the specimen being technically incomplete. The unusual modification and thickening of the exoskeleton indicate that, in spite of a lack of biomineralization, the cuticle was likely hardened by polymers of chitin and other proteins.

Supplementary Discussion

Neural tissues in *Lyrarapax*. Cong and colleagues [5] introduced a new radiodontan from the Chengjiang biota in China with compact body and atypical frontal appendages. They also described carbon-rich patches in the eyes and anterior portion of the head. A single specimen (YKLP 13305) preserves such patches at the very front of the head that the authors interpreted as nerves originating from the protocerebrum and innervating the frontal appendages. Good candidates for arthropod nerves have now been described in several taxa from either the Burgess Shale [6, 7] or the Chengjiang Lagerstätten [8], and, as nerves, consistently preserve as filaments running through podomeres or body segments. In the case of *Lyrarapax*, the features documented are large, bulbous, and have delimited extents on both left and right sides, strongly suggesting they do not project further into the appendages but remain in or overlap their bases. These structures thus clearly differ from known preserved nerves in BST deposits. Being internal, prominent, paired and projecting from an anteriormost carbon patch, there is however little doubt that they are organs related to the central nervous system. It is also important to note that they are located underneath the rounded frontal sclerite, which is slightly displaced anteriad in YKLP 13305. A much more plausible interpretation to us is therefore that these structures are sensory organs (and their tracts) of the inter-ocular complex as described in many early euarthropods [7, 9-11]. Alternatively, based on the relief patterns documented in their Fig. 2b, as well as the margin delimitations of the overall frontal structures, it is also possible that these traces are simply more reflective parts of the central ocular tract, with posterior carbon imprints related to the frontal appendages and/or the mouth. The absence of replication of this evidence in the form of additional specimens renders a final assessment difficult.

Brain morphology in cf. *Alalcomenaeus*. Tanaka et al. [12] provided a detailed description of internal remains in a small leanchoiliid possibly related to *Alalcomenaeus*. It is clear from the distribution of pyrite analyzed both through the elemental mapping of Fe and Micro-CT scanning that the traces highlighted in YKLP 11075 are overlapping features from the outer cuticle, the appendages and internal organs including the gut and, possibly, the CNS as well as other tissues originally compressed into the same kerogen layer. There is no clear rationale to explain why the mapping of iron and the Micro-CT (with these specific settings) could have isolated the CNS better than other elements or methods. The lack of redundancy for such evidence points to a possible taphonomical bias. While neural tissues may well be preserved in this specimen, it has not been clearly discriminated from other tissues, most probably because it is simply not possible based on an original dorso-ventral compression followed by non-

specific replacement by clay minerals and pyrite. Further tentative interpretations regarding the morphology of the brain are thus equally dubious.

Supplementary references

1. Aria C: **Reviewing the bases for a nomenclatural uniformization of the highest taxonomic levels in arthropods**. *Geological Magazine* 2019, **156**(8):1463-1468.
2. Hou X, Bergström J: **Three additional arthropods from the Early Cambrian Chengjiang Fauna, Yunnan, Southwest China**. *Acta Palaleontologica Sinica* 1998, **37**(4):395-401.
3. Strausfeld NJ, Ma X, Edgecombe GD, Fortey RA, Land MF, Liu Y, Cong P, Hou X: **Arthropod eyes: The early Cambrian fossil record and divergent evolution of visual systems**. *Arthropod Structure & Development* 2016, **45**(2):152-172.
4. Hou XG, Siveter DJ, Siveter DJ, Aldridge RJ, Cong P, Gabbott SE, Ma X, Purnell MA, Williams M: **The Cambrian fossils of Chengjiang, China: the flowering of early animal life**. Oxford: Blackwell; 2017.
5. Cong P, Ma X, Hou X, Edgecombe GD, Strausfeld NJ: **Brain structure resolves the segmental affinity of anomalocaridid appendages**. *Nature* 2014, **513**:538-542.
6. Aria C, Caron J-B, Gaines R: **A large new leanchoiliid from the Burgess Shale and the influence of inapplicable states on stem arthropod phylogeny**. *Palaeontology* 2015, **58**(4):629-660.
7. Vannier J, Aria C, Taylor RS, Caron J-B: ***Waptia fieldensis* Walcott, a mandibulate arthropod from the middle Cambrian Burgess Shale**. *Royal Society Open Science* 2018, **5**.
8. Yang J, Ortega-Hernández J, Butterfield NJ, Liu Y, Boyan GS, Hou J-b, Lan T, Zhang X-g: **Fuxianhuiid ventral nerve cord and early nervous system evolution in Panarthropoda**. *Proceedings of the National Academy of Sciences of the United States of America* 2016, **113**(11):2988-2993.
9. Ortega-Hernández J: **Homology of head sclerites in Burgess Shale euarthropods**. *Current Biology* 2015, **25**(12):1625-1631.
10. Hou XG, Bergström J: **Arthropods of the Lower Cambrian Chengjiang fauna, southwest China**. *Fossils and Strata* 1997, **45**:1-116.
11. Aria C, Caron J-B: **Burgess Shale fossils illustrate the origin of the mandibulate body plan**. *Nature* 2017, **545**:89-92.
12. Tanaka G, Hou X, Ma X, Edgecombe GD, Strausfeld NJ: **Chelicerate neural ground pattern in a Cambrian great appendage arthropod**. *Nature* 2013, **502**(7471):364-367.

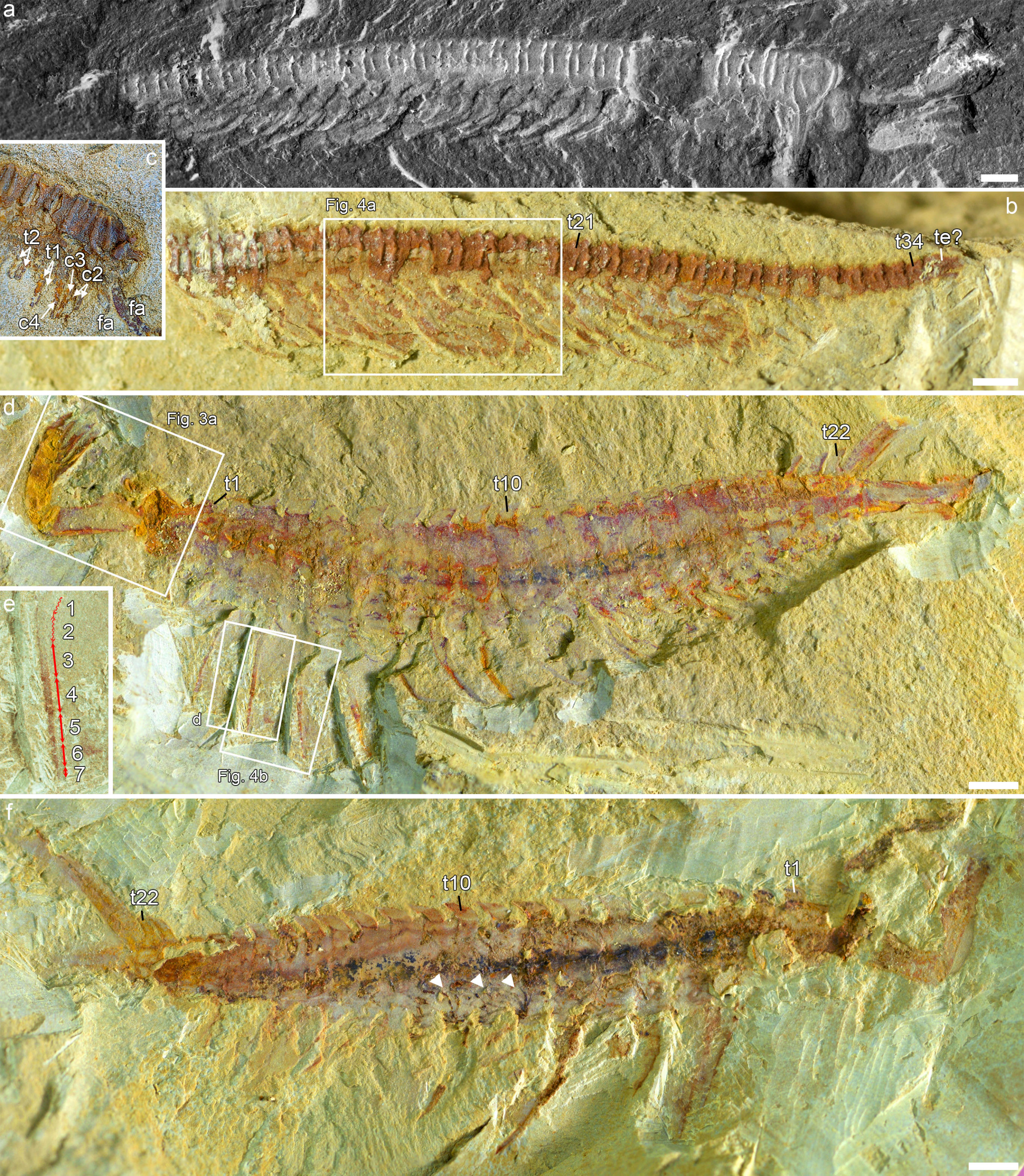
a
b
c
Fig. 4a
t21
t34
te?
t2
t1
c3
c2
c4
fa
fa
d
Fig. 3a
t1
t10
t22
e
1
2
3
4
5
6
7
d
Fig. 4b
f
t22
t10
t1

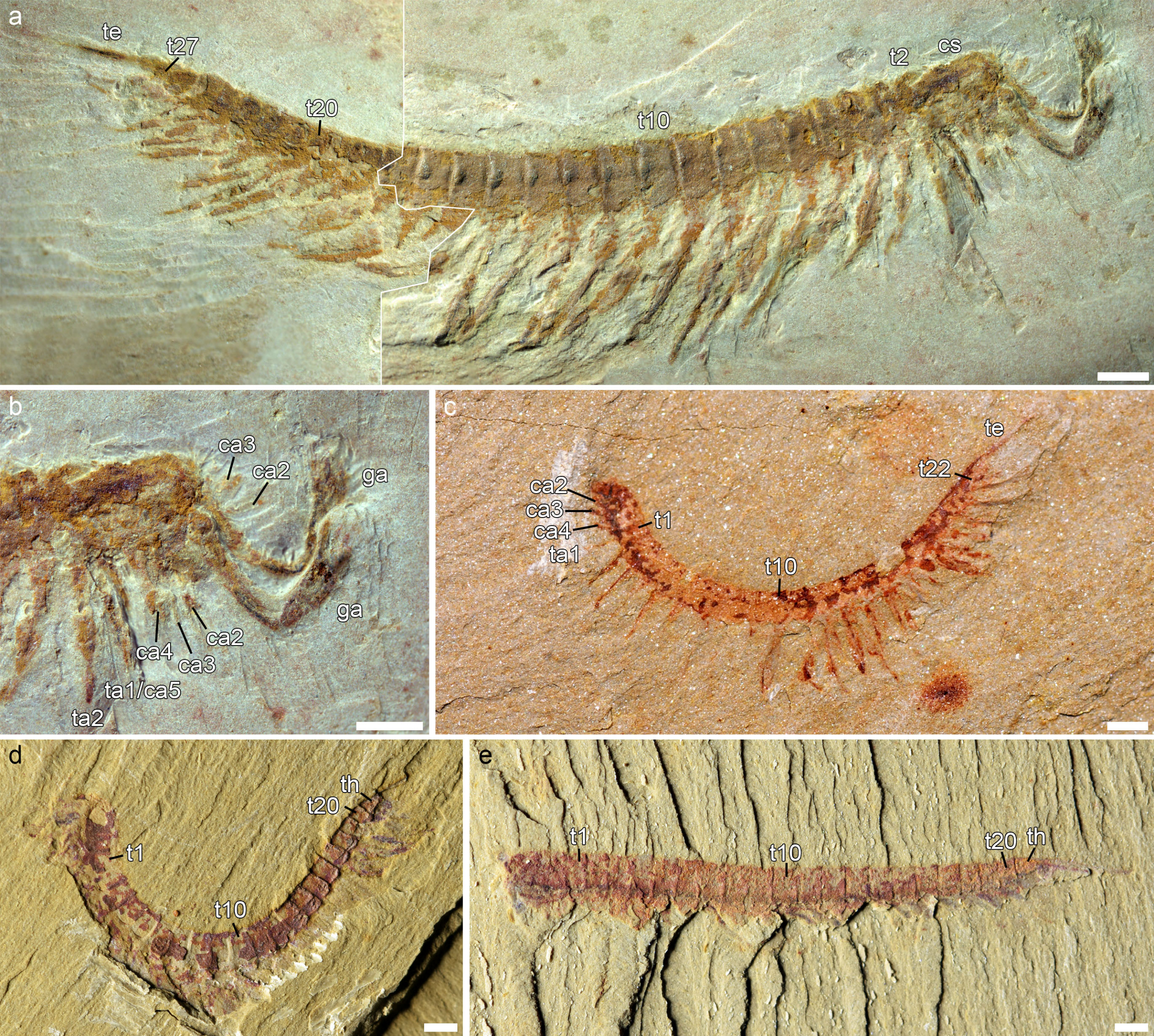
a
te
t27
t20
t10
t2
cs
b
ca3
ca2
ga
ga
ca4
ca3
ca2
ta1/ca5
ta2
c
te
t22
ca2
ca3
ca4
ta1
t1
t10
d
th
t20
t1
t10
e
t1
t10
t20
th

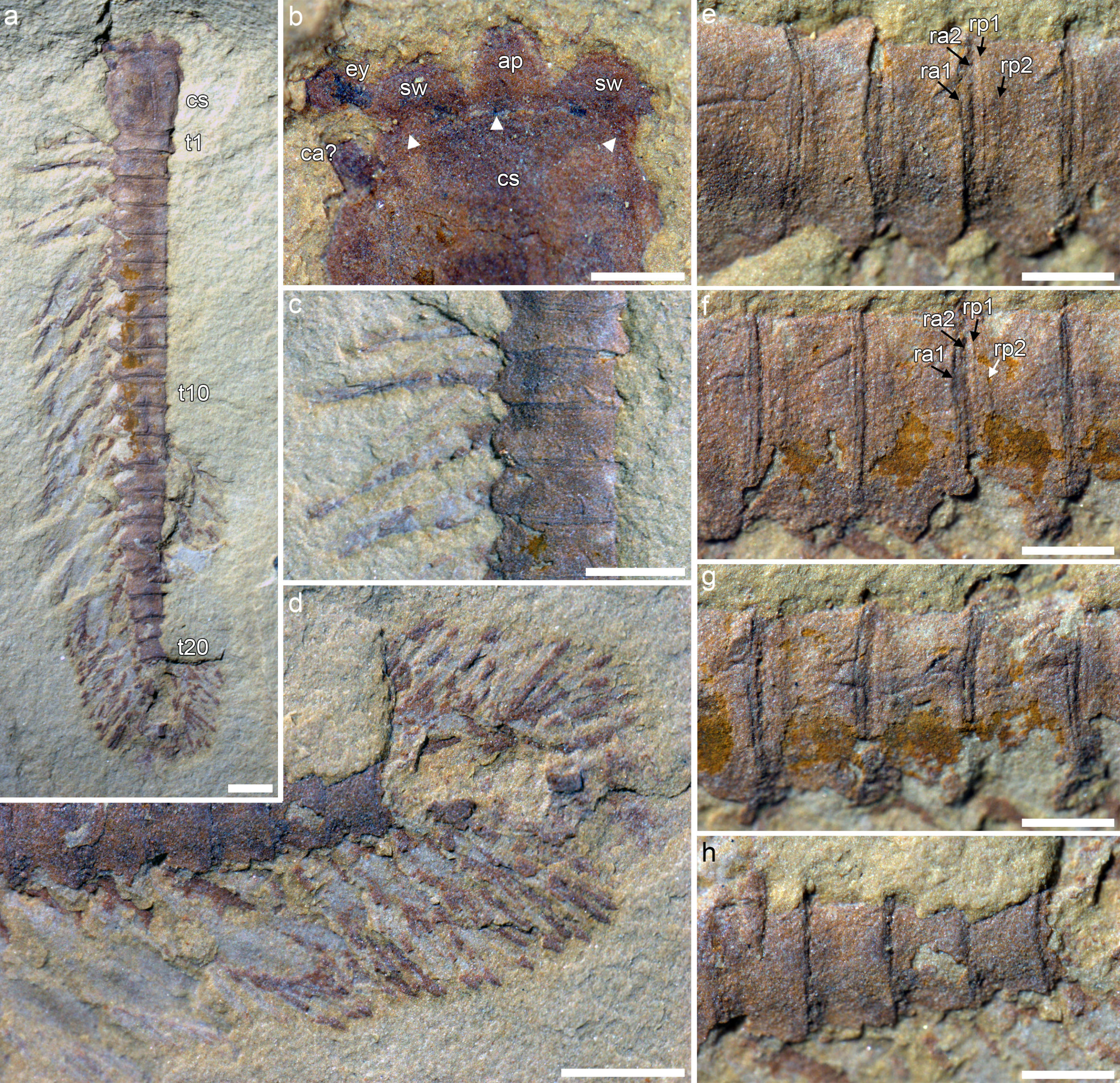

a
cs
t1
t10
t20
b
ey
sw
ap
sw
ca?
cs
c
d
e
ra2
rp1
ra1
rp2
f
ra2
rp1
ra1
rp2
g
h

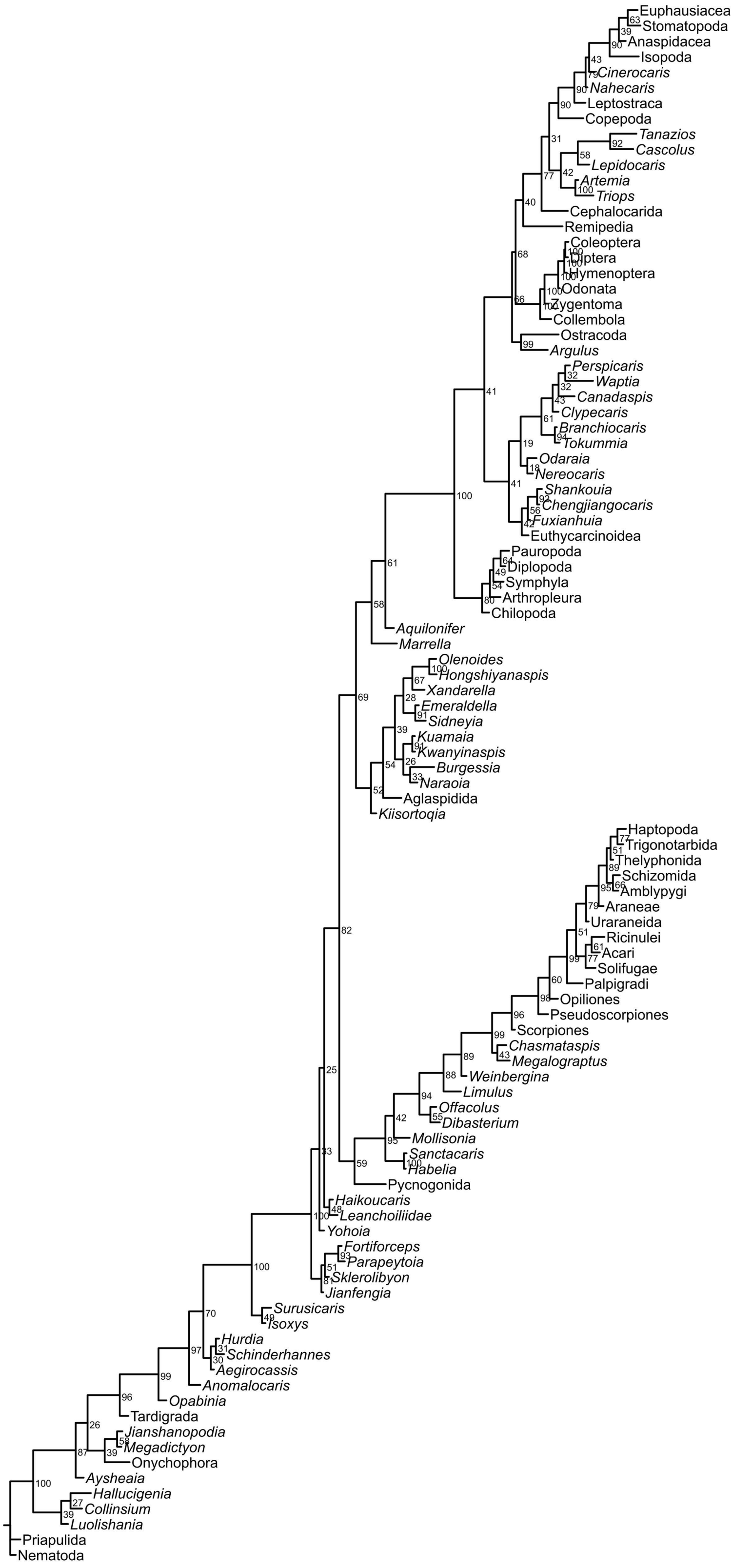
Euphausiacea
Stomatopoda
Anaspidacea
Isopoda
Cinerocaris
Nahecaris
Leptostraca
Copepoda
Tanazios
Cascolus
Lepidocaris
Artemia
Triops
Cephalocarida
Remipedia
Coleoptera
Diptera
Hymenoptera
Odonata
Zygentoma
Collembola
Ostracoda
Argulus
Perspicaris
Waptia
Canadaspis
Clypecaris
Branchiocaris
Tokummia
Odaraia
Nereocaris
Shankouia
Chengjiangocaris
Fuxianhuia
Euthycarcinoidea
Pauropoda
Diplopoda
Symphyla
Arthropleura
Chilopoda
Aquilonifer
Marrella
Olenoides
Hongshiyanaspis
Xandarella
Emeraldella
Sidneyia
Kuamaia
Kwanyinaspis
Burgessia
Naraoia
Aglaspidida
Kiisortoqia
Haptopoda
Trigonotarbida
Thelyphonida
Schizomida
Amblypygi
Araneae
Uraraneida
Ricinulei
Acari
Solifugae
Palpigradi
Opiliones
Pseudoscorpiones
Scorpiones
Chasmataspis
Megalograptus
Weinbergina
Limulus
Offacolus
Dibasterium
Mollisonia
Sanctacaris
Habelia
Pycnogonida
Haikoucaris
Leanchoiliidae
Yohoia
Fortiforceps
Parapeytoia
Sklerolibyon
Jianfengia
Surusicaris
Isoxys
Hurdia
Schinderhannes
Aegirocassis
Anomalocaris
Opabinia
Tardigrada
Jianshanopodia
Megadictyon
Onychophora
Aysheaia
Hallucigenia
Collinsium
Luolishania
Priapulida
Nematoda
