# Supplementary material for: Fossils from South China redefine the ancestral euarthropod body plan
Source: BMC Evol Biol. 2020 Jan 8;20:4. doi: 10.1186/s12862-019-1560-7 (PMC6950928; doi:10.1186/s12862-019-1560-7)
Supplement: Supplementary file 3 — Additional file 3. List of characters used in our morphological matrix. [file 12862_2019_1560_MOESM3_ESM.docx]

**CHARACTER LIST**

The following list is taken from Aria and Caron (2017a) and includes the changes made for the present study as well as those from Aria and Caron (in press). Character headings refer to the original publication by [ACX], where X is the corresponding number for that character in Aria and Caron (2017a). Remarks are not carried over here if no change was made. Characters either new since Aria and Caron (2017), or which have gone through substantial change in overall coding or definition are marked with an asterisk. Some other small corrections were applied to the original matrix but are not reported here in detail.

**GENERAL CHARACTERS**

1. Limbs [AC1]
2. Absent
3. Present
4. External cuticular segmentation [AC2]
5. Absent
6. Present
7. Type of body segmentation [AC3]
8. Sclerotized
9. Arthrodized (=tergal)
10. Calcified cuticle [AC4]
11. Absent
12. Present
13. Visual surface with calcified lenses, bounded by circumocular suture
14. Absent
15. Present

Remark: See Mayers et al. (2018, ch. 12).

1. Holometaboly [AC6]
2. Absent
3. Present

**LOBOPODIAN CHARACTERS**

1. External anteriorization restricted to a single pair of frontalmost appendages [AC7]
2. Absent
3. Present
4. Lobopodous limbs [AC8]
5. Absent
6. Present
7. Type of main lobopodous trunk limb [AC9]
8. Short, conical, subequal or shorter than trunk width
9. Elongated, slender, longer than trunk width
10. Flap-like lateral limbs [AC10]
11. Absent
12. Present
13. Nodes/tubercles/dermal papillae [AC11]
14. Absent
15. Present
16. Differentiation at limb insertion [AC12]
17. Absent
18. Present
19. Dorso-lateral sclerites above limb insertion [AC13]
20. Absent
21. Present
22. Median spine above limb insertions [AC14]
23. Absent
24. Present
25. Lobopod tip (main trunk limb) [AC15]
26. Double claw
27. Juxtaposed series of claws
28. Pad
29. Posterior-most single claws [AC16]
30. Absent
31. Present
32. Posterior claws pointing anteriad [AC17]
33. Absent
34. Present

**VISUAL ORGANS**

1. Ocelli as primary ocular units [AC18]
2. Absent
3. Present
4. Median eyes [AC19]
5. Absent
6. Present
7. Number of median eyes [AC20]
8. 2
9. 3
10. 4
11. Rhabdomeric lateral eye [AC21]
12. Very reduced or absent
13. Present and well developed
14. Type of lateral eyes [AC22]*
15. Few and simple lenses with cup-shaped retina
16. Faceted (compound)
17. Stemmata
18. Type of corneagenous cells [AC23]
19. Many
20. Two
21. Tetraconate condition [AC24]
22. Absent
23. Present
24. Number of nested optic neuropils [AC25]
25. 1
26. 2
27. 3
28. Multi-layered rhabdomeres [AC26]
29. Absent
30. Present
31. Star-shaped rhabdomeres [AC26]
32. Absent
33. Present
34. Lateral compound eye topology [AC27]*
35. Projecting laterally beneath the head shield or through a notch
36. Projecting dorsally and embedded within the shield
37. Projecting latero-ventrally close to the medial axis, possibly accommodated with bulges in shield

Remark: See Mayers et al. (2018, ch. 11).

1. Opthalmic ridges [AC28]
2. Absent
3. Present
4. Lateral eyes pedunculate [AC29]
5. Absent
6. Present
7. Peduncular lobes [AC67]
8. Absent
9. Present

Remark: See Vannier et al. (2018) for a discussion of that character.

1. Pedunculate eyes large and ovate, part of a prominent ocular segment projecting anteriad [AC30]
2. Absent
3. Present

**HEAD AND CEPHALIC CHARACTERS**

1. Somital head (as tagma I) defined by series of appendages and/or external segmentation [AC31]
2. Absent (only anteriormost defines head)
3. Present
4. Somites defining anteriormost tagma* [AC31]
5. 4
6. 5
7. 6
8. 7
9. 8

Remark: Habeliidans and relevant synziphosurines are coded for octasomitic heads following Aria and Caron (2017b).

1. Tergite of the ocular (protocerebral) somite [AC127]*
2. Absent
3. Present

Remark: We consider here that the chelicerate epistome is homologous to other types of frontal sclerites among euarthropods.

1. Tergite of the ocular (protocerebral) somite, type [AC128]
2. Rounded
3. Sub-triangular
4. Supernumerary frontal sclerite attached to the ocular tergite [AC128]
5. Absent
6. Present

Remark: This character codes for the so-called epipharyngeal sclerite attached to the epistome in some chelicerates.

1. Tergal sclerotization of the post-ocular somite [AC33]
2. Absent
3. Present
4. Tergal sclerotization type [AC34]
5. Tergites with posterior expansion over at least some trunk segments (carapace)
6. Tergites with limited expansion, cephalic tergites all fused and articulating or connecting with first trunk segment (shield)
7. Carapace type [AC35]
8. Bivalved
9. Plate
10. Bivalved carapace type*
11. Type I: Sub-straight cross-section, covering body dorsally
12. Type II: Convex cross-section, enveloping body laterally
13. Shape of carapacal valves*
14. Symmetrical respective to sagittal axis, ventral margins tight (‘*Isoxys* type’)
15. Asymmetrical respective to sagittal axis, antero-ventral margin tight, postero-ventral margin ample (‘*Canadaspis* type’)
16. Symmetrical respective to sagittal axis, ventral margins ample (‘*Branchiocaris* type’)
17. Variable (ostracods)
18. Ventral closure of carapace*
19. Absent
20. Present

Remark: ‘Odaraiids’ (*Odaraia* and *Nereocaris*) are characterized by carapacal valves that fully enclose the body, with ventral margins extensively covering the underside of the body. We recognize a similar condition in ostracods.

1. Type II bivalved configuration [AC36]*
2. Unfused along most of dorsal margin
3. Fused along most of dorsal margin

Remark: Among hymenocarines, we consider that *Odaraia* and *Waptia* present an extensive fusion of the valves with effacement of the median fold.

1. Covering of the type II bivalved carapace (when body fully extended antero-posteriorly) [AC37]
2. At least two thirds of body length
3. Cephalothorax
4. Frontal and lateral extensions of cephalic shield*
5. Limited; tergites not raised or expanding laterally
6. Distinct and well-developed; shield forms anterior and/or lateral extensions
7. Articulation of posterior margin of shield with first trunk segment [AC39]
8. Tergal overlap
9. Occipital closure
10. Segmental impression in shield [AC40]
11. Absent
12. Present
13. Occipital lobe [AC41]
14. Absent
15. Present
16. Pair of occipital carinae [AC42]
17. Absent
18. Present
19. Anterior reduction of segments and/or appendages [AC43]
20. Absent
21. Present
22. Compaction of the cephalic unit [AC44]
23. Absent
24. Present
25. Cephalic doublure [AC45]
26. Absent
27. Present
28. Cephalic kinesis [AC46]
29. Absent
30. Present
31. Well-developed pair of genal spines on cephalic shield
32. Absent
33. Present

Remark: We code this character as present in Sklerolibyon, although the spines extend from the fused plate of the first two segments of the shield, and not from

1. Cephalic shield overlapping first trunk sternite, pair of trunk appendages, or trunk caeca
2. Absent
3. Overlap of sixth body somite
4. Overlap of eighth body somite

Remark: See Mayers et al. (2018, ch. 22). This is a multistate character without corresponding sovereign character because states 1 and 2 are unlikely to have a common origin.

1. Dorsal facial sutures
2. Absent
3. Present

**BRAIN CHARACTERS**

1. Ganglia of post-oral cephalic appendages fused into single nerve mass [AC47]
2. Absent
3. Present
4. Trunk ganglia individually expressed*
5. Absent
6. Present

Remark: Generalized from Wang et al. (2018), ch. 146.

1. Contiguity of the first two post-protocerebral ganglia [AC48]
2. Absent
3. Present
4. Fan-shaped body in brain [AC49]
5. Absent
6. Present

1. Position of midline neuropil [AC50]
2. Superficial to protocerebrum
3. Embedded within protocerebral matrix
4. Olfactory lobes linked to a lateral component of protocerebrum by olfactory globular tract [AC51]
5. Absent
6. Present
7. Deutocerebral olfactory lobe with glomeruli [AC52]
8. Absent
9. Present
10. Lateral eyes pedunculated [AC53]
11. Absent
12. Present

**STERNITES (CEPHALON)**

1. Sternites [AC54]
2. Absent
3. Present
4. Endosternum (or endosternite) [AC55]
5. Absent
6. Present
7. Labrum*
8. Absent
9. Present

Remark: Characters describing the hypostome-labrum complex have been reorganized and expanded compared to Aria and Caron (2017). Here, we code the labrum as a common feature shared by all euarthropods, which arguably originates from the protocerebral somite (Scholtz & Edgecombe 2006). Classically, in mandibulates, the “labrum” covers the mouth and such pre-oral structure generally takes two forms: the typical fleshy protrusion of oligostracans, anostracans and other crustaceans, and the sclerotic plates encountered in myriapods, malacostracans and hexapods—which are commonly designated as either “epistome-labrum” or “clypeo-labrum” depending on the group of interest. Pre-labral sclerites in mandibulates are often distinct from the labrum *per se* and are usually closely associated with the insertion of the antennules. For this reason, the pre-labral sclerite is sometimes referred to as the “hypostome,” a common term from the pre-oral sclerite used among extinct euarthropods, especially trilobites and their allies. Such a terminology has been used for instance for Cephalocarida (Olesen et al 2011) but arguably applies to all other crustaceans, including copepods (Schram 1986), leptostracans (Olesen & Walossek 2000) and stomatopods (Haug et al 2012a). Herein (chars. 62-64), we therefore harmonize the terms “epistome,” “clypeus” and “hypostome” under the same concept of “hypostome.”

1. Labrum expression and location*
2. Expressed frontally
3. Expressed as a postero-ventral structure separate from frontalmost organs

Remark: This character fundamentally distinguishes the anteriormost chelicerate “epistome-labrum” from ventral structures characteristic of Mandibulata. Hymenocarines and ostracods are considered to belong to the first group (see Vannier et al., 2018).

1. External inter-ocular sensory organs [AC68]*
2. Absent
3. Present

Remark: Ortega-Hernández and Budd (2016) have discussed their views on the analogous nature of sensory organs and paired inter-ocular projections in panarthropods. Our aim here is to test local cases of homology, and we therefore code this character as present when known. We do however make a distinction for the limb-like inter-ocular features of Canadaspis and remipedes (see char. 86).

1. Type of ventral labrum* [AC58]
2. Single fleshy protrusion
3. Plate with underlying soft tissues
4. Labral plate, type*
5. Bilobate lip with partial fusion to the hypostome
6. Free bilobate or wide lip
7. Expanded subtriangular or subrectangular lip

Remark: This character codes for variations in labral shape amongst mandibulates. Most crustaceans possess state 2, whereas myriapods possess state 0. State 1 is derived and found in pterygotes.

1. Hypostome [AC56]*
2. Absent
3. Present

Remark: The hypostome is here defined on the basis of the artiopodan hypostome, that is, as a pre-oral sclerotic structure associated with the insertion of the anteriormost appendages.

1. Hypostome attachment [AC57]*
2. Conterminant
3. Natant
4. Hypostome accommodating antennules and extensively covering the mouth [AC59]
5. Absent
6. Present

Remark: This is a character mostly discriminating the typical artiopodan hypostome.

1. Mandibulate labium [AC60]
2. Absent
3. Present
4. Chelicerate labium*
5. Absent
6. Present

Remark: The chelicerate labium is a ventral sclerite (sternapophysis) apparently associated with the palpal somite and forming the posterior border of the stomodaeum. We follow Wang et al. (2018) in coding the labium as absent in Pedipalpi but otherwise code state 1 for pseudoscorpions and solifuges after Schultz (2007).

1. Sternites externally developed within segments 2–4 [AC61]
2. Absent
3. Present
4. Fusion of sternites within segments 2–4 [AC62]
5. Absent
6. Present
7. Metastoma [AC63]*
8. Absent
9. Present

Remark: The metastoma is a modified sternite of the first opisthosomal segment in euchelicerates—typically, in eurypterids (Dunlop 1997). We also code this character for scorpions (sternum) and harvestmen (aculi genitales) (Dunlop & Lamsdell 2016).

1. Coxosternite [AC64]
2. Absent
3. Present
4. Single main maxilliped [AC163]
5. Absent
6. Present
7. Head has tendention to form a hypostomal bridge [AC65]
8. Absent
9. Present
10. First opisthosomal tergite triangular
11. Absent
12. Present

Remark: See Wang et al. (2018), ch. 109. Only applies to the total-group Chelicerata given that the homology of this somite requires a prosoma.

1. Genital operculum overlaps sternite of third opisthosomal segment
2. Absent
3. Present

Remark: See Wang et al. (2018), ch. 123. Only applies to the total-group Chelicerata given that the homology of this somite requires a prosoma.

**FRONTALMOST APPENDAGES**

1. Frontalmost limb-like projections
2. Absent
3. Present

Remark: This character codes specifically for the appendicular outgrowths present between the eyes of *Canadaspis*, and comparable ones in remipedes. Their nature and homology with other known cephalic features remain uncertain.

1. Arthrodization of first axial appendage [AC66]
2. Absent
3. Present
4. Size of frontalmost appendage [AC74]
5. Long, multipodomerous; > 6 podomeres
6. Short; ≤ 6 podomeres
7. Well-developed ventral spinose outgrowths on frontalmost appendage [AC76]
8. Absent
9. Present
10. Ventral spinose outgrowths forming elongate rami on frontalmost appendage [AC69]
11. Absent
12. Present
13. Rami of branching frontalmost appendage originating from different podomeres [AC70]
14. Absent
15. Present
16. Short and ramified frontalmost appendage ending in a raptorial device made of three to four elongate spines*
17. Absent
18. Present

Remark: This is a potential apomorphy of Megacheira.

1. Spine morphology of megacheiran appendage*
2. Thin, ca. 10 times as long as thin (base width) or more
3. Thick, ca. 7 times as long as thin (base width) or less
4. Multichelate device type on megacheiran appendage*
5. Made of four spines (‘yohoiid type’)
6. Made of three spines (‘leanchoiliid type’)
7. Podomere number of articulating basis of megacheiran appendage*
8. Single elongate podomere
9. Two short podomeres
10. Single-podomere basis of megacheiran appendage, type*
11. Slender
12. Stout

Remark: *Fortiforceps* and apparently also *Parapeytoia* possess a stouter basal podomere compared to other megacheirans with yohoiid type of “great appendages.”

1. Shape of peduncular podomere for yohoiid great appendages*
2. Sub-cylindrical
3. Chalice-shaped
4. Thickness of peduncular podomere for yohoiid great appendages*
5. Slender
6. Stout

Remark: *Yohoia* and *Jianfengia* have slenderer peduncles compared to other megacheirans. A *Yohoia* morph was reported (Haug et al 2012b) to have stouter ‘great appendages,’ but appears less common—it may also be a different species than *Yohoia tenuis*.

1. Ramified frontalmost appendage with flagellate extensions [AC71]
2. Absent
3. Present
4. Short frontalmost appendage with first and second podomere forming an elbowed articulation*
5. Absent
6. Present

Remark: Typical of non-leanchoiliid megacheirans and some chelicerates, see Haug et al. (2012b) and Wang et al. (2018), ch. 37.

1. Frontalmost appendage a chelicera, i.e. chelate or subchelate with only two opposing faces [AC72]
2. Absent
3. Present
4. Orientation of closure of terminal podomere (apotele) on chelicera*
5. Ventral
6. Dorsal
7. Lateral

Remark: See Wang et al. (2018), ch. 40.

1. Cheliceral fang*
2. Absent
3. Present

Remark: See Wang et al. (2018), ch. 41.

1. Number of cheliceral podomeres*
2. Three
3. Two

Remark: See Wang et al. (2018), ch. 35.

1. Cheliceral serrula*
2. Absent
3. Present

Remark: See Wang et al. (2018), ch. 53.

1. Orientation of first axial appendage [AC73]
2. Ventro-frontal
3. Dorsal
4. Arthrodized frontalmost appendage, multipodomerous type [AC75]
5. Robust, thick branch
6. Long antennular
7. Type of inner (ventral) spinose outgrowths on frontalmost appendage [AC77]
8. Sub-equal length or tapering gradually along entire margin
9. Elongate mid-margin
10. Secondary spines on ventral spinose outgrowths of frontalmost appendage [AC78]
11. Absent
12. Present
13. Dorsal spinose outgrowths on podomeres of arthrodized frontalmost appendage [AC79]
14. Absent
15. Present

**OTHER CEPHALIC LIMBS**

1. Maximum podomere number in head (tagma I) [AC90]*
2. 7
3. <7
4. >7

Remark: When endopods are differentiated and vary from one somite to the other, this character takes into account the endopod with the highest number of podomeres.

1. Punctual subdivision of endopod podomeres from an heptopodomeran limb*
2. Absent
3. Basi- and telofemur
4. Basi- and telotarsus

Remark: See Wang et al. (2018), chs. 88 and 93. This multistate has no sovereign character because states 1 and 2 do not necessarily have a common origin.

1. All cephalic endopods posterior to frontalmost appendage pair well-developed (seven-segmented or more) [AC81]*
2. Absent
3. Present

Remark: Taking the heptopodomeran condition as a reference for the ground pattern (see Aria et al. 2015), endopods are considered reduced here when the podomere count is below seven. Sometimes an exact podomere count has not been possible so far (e.g. in the first post-antennular limb of leanchoiliids) and the modified state is occasionally extrapolated from a substantial reduction of the appendage in size.

1. Endopod of second appendage pair [AC82]
2. Developed
3. Reduced
4. Endopod of third appendage pair [AC83]
5. Developed
6. Reduced
7. Endopod of fourth appendage pair [AC84]
8. Developed
9. Reduced
10. Exopod of fourth appendage pair [AC112]*
11. Developed
12. Reduced
13. Endopod of fifth appendage pair [AC85]
14. Developed
15. Reduced
16. Exopods of fifth appendage pair*
17. Developed
18. Reduced
19. Exopods on cephalic appendages posterior to fifth pair*
20. Absent
21. Present
22. Exopod of cephalic appendages excluding two anteriormost pairs, type [AC102]
23. Stenopodous, podomeres > 4
24. Annulate
25. Short, few podomeres (≤ 4)

Remark: See Mayers et al. (2018).

1. Subdivision of short cephalic exopod, type
2. Bipartite
3. Tripartite

Remark: See Mayers et al. (2018).

1. Stenopodous exopod, type [AC103]
2. Antenniform with elongate podomeres
3. Short and stout, ending in setal brush (underdeveloped claw)
4. Partial detachment of exopods from main limb branch in head tagma*
5. Absent
6. Present

Remark: This character expresses the peculiar condition of habeliidans, *Offacolus* and *Dibasterium*, in which the cephalic exopods preserve as partially dissociated from their main biramous branch (Aria & Caron 2017b). The exact attachment remains unknown.

1. Some cephalic endopods are walking limbs [AC86]
2. Absent
3. Present
4. Repeated appendage morphology in tagma I [AC87]
5. Absent
6. Present
7. Dichotomy in appendage morphology between tagma I and tagma II [AC88]
8. Absent
9. Present
10. Proximo-distal differentiation of endopod podomeres in head (tagma I) [AC89]
11. Absent
12. Present
13. Arthrodized post-antennular appendage expressed [AC91]
14. Absent
15. Present
16. Post-antennular appendage differentiated [AC92]
17. Absent
18. Present
19. Chelate or sub-chelate termination of post-antennular appendage [AC93]*
20. Absent
21. Present
22. Plane of motion of chelate or sub-chelate post-antennular appendage*
23. Sub-vertical, leg-like
24. Horizontal

Remark: See Wang et al. (2018), ch. 62.

1. Ramification of post-antennular appendage [AC95]
2. Uniramous
3. Biramous
4. Developed endites on endopod of post-antennular appendage [AC96]
5. Absent
6. Present
7. Endopod of post-antennular appendage annulate or flagellate [AC97]
8. Absent
9. Present
10. Coxa on post-antennular appendage [AC99]
11. Absent
12. Present
13. Exopod of post-antennular appendage, type [AC100]
14. Stenopodous
15. Annulate
16. Short, lobate
17. Endopod of third cephalic appendage very thin and elongate, filament-like*
18. Absent
19. Present

Remark: This condition is sometimes called “antenniform” in chelicerates (e.g. Dunlop and Lamsdell 2016). We differentiate the chelicerate condition from the annulate condition, that is, with podomeres extremely thin and elongate, as opposed to being very short and numerous.

1. Enditic outgrowths on cephalic endopods excluding two anteriormost pairs [AC104]
2. Absent
3. Present
4. Endopod of third cephalic appendage chelate or subchelate [AC105]
5. Absent
6. Present
7. Third cephalic appendage with a well-developed, toothed gnathobase [AC106]
8. Absent
9. Present
10. Third cephalic appendage a mandible [AC107]
11. Absent
12. Present
13. Mandibular palp [AC107]
14. Non-developed
15. Developed
16. Mandible with three-segmented palp, appressed on the ventral side of the head, curving inward [AC94]*
17. Absent
18. Present

Remark: This characterizes the endopod of the fuxianhuiid mandible.

1. Telognathic mandible [AC108]
2. Absent
3. Present
4. Mandibular gnathal edge [AC109]
5. Consisting of molar and incisor process
6. Only ellipsoid pars molaris present
7. Row of parallel teeth
8. Shovel with terminal teeth
9. Group of paired teeth and hair pad
10. Mandibular lamellate combs [AC110]
11. Absent
12. Present
13. Hypopharynx [AC111]
14. Absent
15. Present
16. Modified endopod/palp on fourth cephalic appendage [AC113]
17. Absent
18. Present

Remark: This character implies the modification of the appendage basis as a mouthpart and the reduction of the endopod of the fourth appendage pair (char. 90), whereby the complete reduction of the endopod is coded “0”.

1. Modified endopod/palp on fourth cephalic appendage, type [AC114]*
2. Reduced, vestigial, undeveloped
3. Well developed
4. Post-mandibular plate formed by the fusion of the maxilla and the intermaxillary sternum [AC115]
5. Absent
6. Present
7. Cephalic appendages 4 and 5 ending with chelate termination [AC116]
8. Absent
9. Present
10. Fifth cephalic appendage, differentiation type [AC117]
11. Integrated to gnathal plate (labium)
12. Reduced, enditic
13. Fifth cephalic appendage vestigial [AC118]
14. Absent
15. Present
16. Fifth cephalic appendage with developed palp [AC119]
17. Absent
18. Present

Remark: Same requirements for coding as for char. 121, but with respect to fifth cephalic pair.

1. Internalization of mouthparts [AC120]
2. Absent
3. Present
4. Oral cone [AC121]
5. Absent
6. Present

1. Atrium oris [AC122]
2. Absent
3. Present

**MOUTH AND STOMODAEAL AREA**

1. Mouth opening [AC123]
2. Frontal
3. Ventral
4. Ventral mouth opening, type
5. Antero-ventral
6. Postero-ventral
7. Type of circumoral structures [AC124]*
8. Toothed lips
9. Lamellae
10. Ring of plates
11. Mouth surrounded by arthrodized limbs
12. Circumoral structures sclerotized [AC125]*
13. Absent
14. Present
15. Proboscis [AC126]
16. Absent
17. Present

**ALIMENTARY TRACT AND OTHER INTERNAL CHARACTERS**

1. Large and well-differentiated stomach [AC129]
2. Absent
3. Present
4. Stomach in a frontal position [AC131]
5. Absent
6. Present
7. Stomach—additional pouch (crop) [AC132]
8. Absent
9. Present
10. Secondary organs connected to the central digestive duct [AC132]
11. Absent
12. Present
13. Secondary digestive organs serially repeated along the post-cephalic portion of the gut [AC133]
14. Absent
15. Present
16. Shape of post-cephalic secondary digestive structures [AC134]
17. Reniform
18. Bulgy triangles
19. Caeca
20. Striations on post-cephalic secondary digestive structures [AC135]
21. Absent
22. Present
23. Branching of post-cephalic secondary digestive structures [AC136]
24. Absent
25. Present
26. Differentiation of cephalic secondary digestive structures (compared to trunk) [AC137]
27. Absent
28. Present
29. Ramification of cephalic secondary digestive structures [AC138]
30. Absent
31. Present
32. Branching of cephalic secondary digestive structures [AC139]
33. Absent
34. Present
35. Peritrophic membrane [AC140]
36. Absent
37. Present
38. Metameric ganglia on nerve cord [AC141]
39. Absent
40. Present
41. Metanephridia with sacculus containing podocytes [AC142]
42. Absent
43. Present
44. Segmental invaginations of neuroectoderm giving rise to ventral organs [AC143]
45. Absent
46. Present
47. Malpighian tubules*
48. Absent
49. Present

Remark: See Wang et al. (2018), ch. 164, for coding in arachnids.

1. Coxal glands opening at base of prosomal leg 1*
2. Absent
3. Present

Remark: See Wang et al. (2018), ch. 166. Only applies to Euchelicerata.

1. Distodorsal insertion of posterior transpatellar muscle*
2. Absent
3. Present

Remark: See Wang et al. (2018), ch. 190. Muscle data in chelicerates carry some obvious conflicts, both internally and with other parts of the anatomy. We selected those characters based on musculature that provide information on major internal arachnid subdivisions (aside from the extensive evidence supporting the monophyly of Arachnida and Pedipalpi, methodologically enforced in our analysis) but do not assume spurious configurations, such as a polyphyletic tetrapulmonata. We also kept the information to the homologous insertions of the muscles and did not assume losses as having common origins in case of multistates. Only applies to Euchelicerata.

1. Ventral insertion of the anterior transpatellar muscle*
2. Absent
3. Present

Remark: See Wang et al. (2018), ch. 192. Only applies to Euchelicerata.

1. Ventral insertion of the anterior patellotibial muscle on tibia*
2. Absent
3. Present

Remark: See Wang et al. (2018), ch. 193. Only applies to Euchelicerata.

1. Posterior patellotibial muscle*
2. Absent
3. Present

Remark: See Wang et al. (2018), ch. 194. Only applies to Euchelicerata.

1. 9x2 +3 microtubule arrangement in euchelicerate sperm axoneme*
2. Absent
3. Present

Remark: See Wang et al. (2018), ch. 218. Only applies to Euchelicerata.

1. Iso/telolecithal euchelicerate eggs*
2. Absent
3. Present

Remark: See Wang et al. (2018), ch. 226. Only applies to Euchelicerata.

**TRUNK**

1. Fusion of trunk tergites
2. Absent
3. Present

Remark: See Mayers et al. (2018, ch. 2).

1. Fusion of trunk tergites, type
2. Partial—some segments still freely articulate
3. Complete—all trunk tergites show some degree of fusion

Remark: See Mayers et al. (2018, ch. 3).

1. Degree of effacement of tergite boundaries on fused trunk portion
2. Low—pleurae outstanding, at least laterally
3. High—pleurae margins fused, at least laterally
4. Tergo-pleurae individualized and arthrodial membranes sometimes visible, but with no tergite overlap

Remark: See Mayers et al. (2018, ch. 5). State 2 is added here to reflect the condition of most terrestrial euarthropods. Certain chelicerates (Haptopoda, Uropygi) have fused trunks but retain overlapping tergites.

1. Distinct pygidium [AC209]
2. Absent
3. Present

Remark: See Mayers et al. (2018, ch. 7).

1. Thorax [AC144]
2. Absent
3. Present
4. Number of thoracic somites [AC145]
5. 11+
6. 4/5
7. 7/9
8. 3
9. Abdomen [AC146]
10. Absent
11. Present
12. Post-abdomen
13. Absent
14. Present

Remark: A post-abdomen is defined here by the differentiation of limbless tergo-pleurae posterior to an abdomen.

1. Multisegmentation (trunk somites ≥ 20) [AC149]
2. Absent
3. Present
4. Number of core trunk segments (non multisegmented taxa) [AC147]*
5. 15-19
6. 10-14
7. 9
8. 7-8
9. <7
10. Seventh appendage integrated into the prosoma [AC148]*
11. Absent
12. Present

Remark: This character only applies to Chelicerata and their stem groups (see also char. 32).

1. Tergite of eighth somite (counting the ocular somite as the first) drastically reduced as a “microtergite”*
2. Absent
3. Present

Remark: See Dunlop and Lamsdell (2016) for a review of this character across chelicerates.

1. Constriction of eighth somite (segment seven) into a pedicel*
2. Absent
3. Present

Remark: A widespread arachnid character (see e.g. Dunlop and Lamsdell (2016)).

1. Post-cephalic appendages covered by sclerotic plates (opercula)*
2. Absent
3. Present

Remark: Used as an apomorphy of Euchelicerata (Aria & Caron in press, Dunlop & Lamsdell 2016), although it mostly applies to merostomes and tetrapulmonates.

1. Tergo-sternal decoupling [AC150]
2. Absent
3. Present
4. Tergo-sternal decoupling, type [AC151]
5. Polypody
6. Polypody and “polysternity”
7. “Polytergity” (autapomorphy of symphylan myriapods)
8. Pleurae [AC152]
9. Reduced or fused
10. Developed
11. Tergo-pleural rings [AC153]*
12. Absent
13. Present
14. Pleural orientation [AC154]
15. Horizontal
16. Around body
17. Pleural length [AC155]
18. Short, i.e. equal or inferior to body diameter
19. Long, i.e. exceeding body diameter
20. Articulating ridge [AC156]
21. Absent
22. Present
23. Articulating ridge, type [AC157]
24. Single
25. Antero-posterior
26. Transverse stipital muscle [AC158]
27. Absent
28. Present

**TRUNK APPENDAGES AND GENERAL APPENDICULAR CHARACTERS**

1. Limb arthrodization in trunk [AC181]
2. Absent
3. Present
4. Proximo-distal differentiation of endopod podomeres in tagma II [AC159]
5. Absent
6. Present
7. Podomere number in endopods of tagma II [AC160]
8. 7
9. <7
10. >7
11. Maxillipeds [AC161]
12. Absent
13. Present
14. Tergites of maxilliped segments fused to head shield [AC162]
15. Absent
16. Present
17. Maxilliped pairs*
18. Single main pair
19. Several pairs
20. Slit sensilla [AC164]
21. Absent
22. Present
23. Trichobothria*
24. Absent
25. Present

Remark: See Wang et al. (2018), ch. 139.

1. Basis (basipod) [AC165]
2. Absent
3. Present
4. Basipod formed of at least two elements [AC166]
5. Absent
6. Present
7. Basipod multi-segmented [AC167]
8. Absent
9. Present
10. Multiple endites on basipod [AC168]
11. Absent
12. Present
13. Proximalmost endite on basipod [AC169]
14. Absent
15. Present
16. Coxa as entire pre-basal podomere [AC170]
17. Absent
18. Present
19. Precoxa as whole pre-coxal podomere [AC171]
20. Absent
21. Present
22. Pleurites formed by several sclerotic elements surrounding limb insertion [AC172]
23. Absent
24. Present
25. Arrangement of pleurites [AC173]
26. Outer/proximal and distal/inner sets
27. Multiple sclerotic pieces
28. Gnathobases (= heavily-sclerotized and toothed masticatory basipods) on any body limb [AC174]
29. Absent
30. Present
31. One or more gnathobase(s) reduced in tagma I [AC175]
32. Absent
33. Present
34. Secondary appendicular outgrowths on trunk [AC176]
35. Absent
36. Present
37. Secondary appendicular outgrowths on trunk, type [AC177]
38. Lobopodous
39. Sclerotized
40. Distal oblanceolate lamellae on exopod
41. Absent
42. Present
43. Proximal lamellae (gills) with appendicular affinity in trunk [AC178]
44. Absent
45. Present
46. Proximal lamellae (gills) present on trunk somites 10 to 13*
47. Absent
48. Present

Remark: Generalized from Wang et al. (2018), ch. 152, to all relevant arthropods.

1. Book gills made of a series of broad, overlapping lamellae*
2. Absent
3. Present

Remark: This character codes for the gill form generally characteristic of euchelicerates, worded to also include variations with fewer lamellae than those known in merostomes.

1. Proximal lamellae internalized [AC179]
2. Absent
3. Present
4. Trunk exopod posterior to head tagma, type [AC182]*
5. Reduced, vestigial
6. Annulate
7. Short, few podomeres (≤ 4)
8. Phyllopodous
9. Short trunk exopod, type
10. Single lobe
11. Bipartite
12. Tripartite
13. Trunk endopod reduced posterior to head tagma [AC180]
14. Absent
15. Present
16. Endopod strongly developed in thorax (or anterior trunk if thorax undifferentiated) [AC183]
17. Absent
18. Present
19. Phyllopodous-type limbs anywhere on body [AC184]
20. Absent
21. Present
22. Terminal endopods stenopodous [AC185]
23. Absent
24. Present
25. Identical morphology of endopod and exopod rami on pleopods/post-thorax [AC186]
26. Absent
27. Present
28. Annulation of at least one pair of exopods [AC187]
29. Absent
30. Present
31. Epipod [AC191]
32. Absent
33. Present
34. Endites as latero-distal projections on endopod podomeres [AC192]
35. Absent
36. Present
37. Pusher legs with paddle tips [AC193]
38. Absent
39. Present
40. Developed endites on endopod podomeres in trunk (tagma II and III) [AC194]
41. Absent
42. Present
43. Paired spines on endopod podomeres [AC195]
44. Absent
45. Present
46. Short spines on endopod podomeres [AC196]
47. Absent
48. Present
49. Multiple setae on endopod podomeres [AC197]
50. Absent
51. Present
52. Complex articulation between basipod and first endopod podomere involving surnumerary sclerites*
53. Absent
54. Present

Remark: Generalized from Wang et al. (2018), ch. 87. Mandibulate pleurites are not considered here.

1. Bicondylar femoropatellal articulation*
2. Absent
3. Present

Remark: See Wang et al. (2018), ch. 89. Only applicable to euchelicerates.

1. Patellotibial articulation*
2. Monocondylar
3. Hinge
4. Bicondylar

Remark: See Wang et al. (2018), ch. 90. Only applicable to euchelicerates.

1. Main limb tip along body [AC199]
2. Pad
3. Juxtaposed claws
4. Trident of claws
5. Chelate or sub-chelate
6. Double claw
7. Multiple spines
8. Single claw
9. Coxal vesicles*
10. Absent
11. Present

Remark: So-called “sternal pores” in euthycarcinoids have been reinterpreted by Edgecombe and Morgan (1999) as probable homologs to the coxal vesicles of certain terrestrial mandibulates, including Symphyla and Diplopoda. We follow this view here.

**POSTERIOR TERMINATION**

1. Sclerotization of termination [AC200]
2. Absent
3. Present
4. Telson developed [AC201]
5. Absent
6. Present
7. Telson type [AC202]
8. Spine
9. Plate / Spatula
10. Flagellate extension of telson*
11. Absent
12. Present
13. Anus location [AC203]
14. Terminal somite
15. Base of telson
16. Caudal rami [AC204]
17. Absent
18. Present
19. Caudal rami, type [AC208]
20. Spinose
21. Annulate
22. Subdivided
23. Rounded
24. Flap
25. Additional caudal processes [AC205]
26. Absent
27. Present
28. Furca [AC206]
29. Absent
30. Present
31. Uropods sensu stricto [AC207]
32. Absent
33. Present
34. Axial elevation of pygidium [AC211]
35. Absent
36. Present
37. Pygidial ornamentation [AC212]
38. Smooth
39. Spinose

**REFERENCES**

Aria C, Caron J-B. 2017a. Burgess Shale fossils illustrate the origin of the mandibulate body plan. *Nature* 545: 89-92

Aria C, Caron J-B. 2017b. Mandibulate convergence in an armoured Cambrian stem chelicerate. *BMC Evolutionary Biology* 17: 261

Aria C, Caron J-B. in press. Mandibulate convergence in an armoured Cambrian stem chelicerate. *BMC Evolutionary Biology*

Dunlop JA. 1997. *The origins of tetrapulmonate book lungs and their significance for chelicerate phylogeny*. Presented at Proceedings of the 17th European Colloquium of Arachnology, Edinburgh

Dunlop JA, Lamsdell JC. 2016. Segmentation and tagmosis in Chelicerata. *Arthropod Structure & Development* 46: 395-418

Edgecombe GD, Morgan H. 1999. Synaustrus and the euthycarcinoid puzzle. *Alcheringa* 23: 193-213

Haug C, Sallam WS, Maas A, Waloszek D, Kutschera V, Haug JT. 2012a. Tagmatization in Stomatopoda - reconsidering functional units of modern-day mantis shrimps (Verunipeltata, Hoplocarida) and implications for the interpretation of fossils. *Frontiers in Zoology* 9

Haug JT, Waloszek D, Maas A, Liu Y, Haug C. 2012b. Functional morphology, ontogeny and evolution of mantis shrimp-like predators in the Cambrian. *Palaeontology* 55: 369-99

Olesen J, Haug JT, Maas A, Waloszek D. 2011. External morphology of Lightiella monniotae (Crustacea, Cephalocarida) in the light of Cambrian 'Orsten' crustaceans. *Arthropod Structure & Development* 40: 449-78

Olesen J, Walossek D. 2000. Limb ontogeny and trunk segmentation in Nebalia species (Crustacea, Malacostraca, Leptostraca). *Zoomorphology* 120: 47-64

Ortega-Hernandez J, Budd GE. 2016. The nature of non-appendicular anterior paired projections in Palaeozoic total-group Euarthropoda. *Arthropod Structure & Development* 45: 185-99

Scholtz G, Edgecombe GD. 2006. The evolution of arthropod heads: reconciling morphological, developmental and palaeontological evidence. *Development Genes and Evolution* 216: 395-415

Schram FR. 1986. *Crustacea*: Oxford University Press. 606 pp.

Shultz JW. 2007. A phylogenetic analysis of the arachnid orders based on morphological characters. *Zoological Journal of the Linnean Society* 150: 221-65

Vannier J, Aria C, Taylor RS, Caron J-B. 2018. *Waptia fieldensis* Walcott, a mandibulate arthropod from the middle Cambrian Burgess Shale. *Royal Society Open Science* 5

Wang B, Dunlop JA, Selden PA, Garwood RJ, Shear WA, et al. 2018. Cretaceous arachnid *Chimerarachne yingi* gen. et sp. nov. illuminates spider origins. *Nat Ecol Evol* 2: 614-22
